# The bactericidal and antibiofilm effects of a lysine-substituted hybrid peptide, CM-10K14K, on biofilm-forming *Staphylococcus epidermidis*

**DOI:** 10.1038/s41598-023-49302-y

**Published:** 2023-12-14

**Authors:** Natthaporn Klubthawee, Mathira Wongchai, Ratchaneewan Aunpad

**Affiliations:** 1 Department of Medical Technology, Faculty of Allied Health Sciences, Thammasat University, Khlong Luang, 12120 Pathum Thani Thailand; 2 Graduate Program in Biomedical Sciences, Faculty of Allied Health Sciences, Thammasat University, Khlong Luang, 12120 Pathum Thani Thailand

**Keywords:** Antimicrobials, Microbiology, Drug discovery

## Abstract

Staphylococci, notably biofilm-forming *Staphylococcus epidermidis*, have been recognized as global nosocomial pathogens in medical device-related infections. Their potential to attach to and form biofilm on indwelling catheters are significant factors impeding conventional treatment. Due to their extensive antimicrobial and antibiofilm actions, antimicrobial peptides (AMPs) have attracted interest as promising alternative compounds for curing difficult-to-treat, biofilm-forming bacterial infections. Cecropin A-melittin or CM, a well-known hybrid peptide, exhibits broad-spectrum antimicrobial activity, however it also possesses high toxicity. In the current study, a series of hybrid CM derivatives was designed using an amino acid substitution strategy to explore potential antibacterial and antibiofilm peptides with low toxicity. Among the derivatives, CM-10K14K showed the least hemolysis along with potent antibacterial activity against biofilm-forming *S. epidermidis* (MICs = 3.91 μg/mL) and rapid killing after 15 min exposure (MBCs = 7.81 μg/mL**)**. It can prevent the formation of *S. epidermidis* biofilm and also exhibited a dose-dependent eradication activity on mature or established *S. epidermidis* biofilm. In addition, it decreased the development of biofilm by surviving bacteria, and formation of biofilm on the surface of CM-10K14K-impregnated catheters. Released CM-10K14K decreased planktonic bacterial growth and inhibited biofilm formation by *S. epidermidis* in a dose-dependent manner for 6 and 24 h post-exposure. Impregnation of CM-10K14K prevented bacterial attachment on catheters and thus decreased formation of extensive biofilms. SEM images supported the antibiofilm activity of CM-10K14K. Flow cytometry analysis and TEM images demonstrated a membrane-active mechanism of CM-10K14K, inducing depolarization and permeabilization, and subsequent membrane rupture leading to cell death. The presence of an interaction with bacterial DNA was verified by gel retardation assay. These antibacterial and antibiofilm activities of CM-10K14K suggest its potential application to urinary catheters for prevention of biofilm-forming colonization or for treatment of medical devices infected with *S. epidermidis*.

## Introduction

*Staphylococcus epidermidis*, an opportunistic biofilm former and a common commensal of the human skin, is the most prevalent of coagulase-negative staphylococcus and the major cause of infection of implanted medical devices^1–3^. *Staphylococcus epidermidis* adhere to the medical devices, then multiply and develop condensed populations covered in a defensive matrix called a biofilm^4^. This matrix makes difficult the treatment of bacteria within this biomass^1^.

The Centers for Disease Control and Prevention (CDC) reported that approximately 75% of hospital-acquired, urinary tract infections (UTIs) are associated with catheters placed through the urethra into the bladder to empty urine. Prolonged catheter use is recognized as one of the major risk factors for the development of catheter-associated urinary tract infections (CAUTIs)^5^, another being immunocompromise of the patient^6^.

There is presently no commercially available technology to prevent CAUTIs^7^. Using closed drainage systems, ensuring sterility at the time of insertion, and minimizing the duration of catheterization are considered best practices^8,9^. More experimental is the incorporation of antimicrobial compounds to release antimicrobial agents, inhibit attachment or kill microbes, and disturbance of the biofilm’s formation in order to prevent or limit UTIs^10^.

Several antimicrobial agents have been explored, such as impregnating the catheter with antibiotics, silver or antiseptics. Belfield and colleagues demonstrated that rifampicin-, triclosan-, and sparfloxacin-impregnated urinary catheters have a long-term preventive effect against key uropathogens (including methicillin-resistant *S. aureus*, methicillin-resistant *S. epidermidis*, extended-spectrum beta-lactamase producing *Escherichia coli*, and carbapenemase-producing *E. coli*)^7^. Johnson and co-workers compared the effectiveness and efficiency of nitrofurazone- and silver-alloy-coated catheters, and found the former is more cost-efficient and effective at preventing the formation of biofilms and planktonic growth^11,12^. Segev et al. showed that urinary catheters coated with a chlorhexidine sustained-release varnish have a significantly reduced development of biofilms^13^. Although these catheters demonstrated good antibacterial action in vitro, their limitations include significant cytotoxicity, short duration of drug release, and development of bacterial resistance restricting their long-term use^14^. Therefore, the search for novel substances is ongoing.

Antimicrobial peptides (AMPs) are short polymers of amino acids with broad-spectrum bactericidal activities and infrequent development of bacterial resistance. In addition, they have been demonstrated to inhibit microbes from colonizing surfaces, kill bacteria in biofilms, and disturb the structure of biofilms^15^. As such, AMPs have potential in the development of alternative antimicrobial catheters^16^. Lim and colleagues demonstrated that the potent antimicrobial and anti-biofilm properties of a catheter with a polydopamine-peptide (CWR11) coating are comparable to those of commercial Dover silver-coated catheters^14^. A polyurethane catheter with a brush coating tethered with AMP E6 reduced bacterial adhesion to the catheter’s surface in a mouse urinary catheter infection model^17,18^. In addition, the lipopeptide GZ3.27 has potential in the antimicrobial coating of medical implants^19^.

In this study, the derivative peptides of a parent cecropin A and melittin hybrid were designed and studied for their antimicrobial and antibiofilm properties, and the mechanisms of these actions. Various methods have previously been employed to coat the surfaces of medical devices; impregnation is an effective and simple method and was used in this study. The candidate peptide-impregnated catheters were then evaluated in bactericidal activity against free-floating (planktonic) *S. epidermidis* and inhibition of biofilm formation adherent to the catheter surface.

## Materials and methods

### Peptide design and in silico analysis

To further develop the parent Cecropin A-Melittin (or CM) hybrid peptide^20^ with dual activities as antibacterial and antibiofilm against *S. epidermidis* biofilm-associated infections on a catheter model, two sets of CM derivatives were designed based on its α-helical secondary structure and helical wheel projection for determining the effect of net charge and hydrophobicity on antibacterial action.

**Figure 1:**
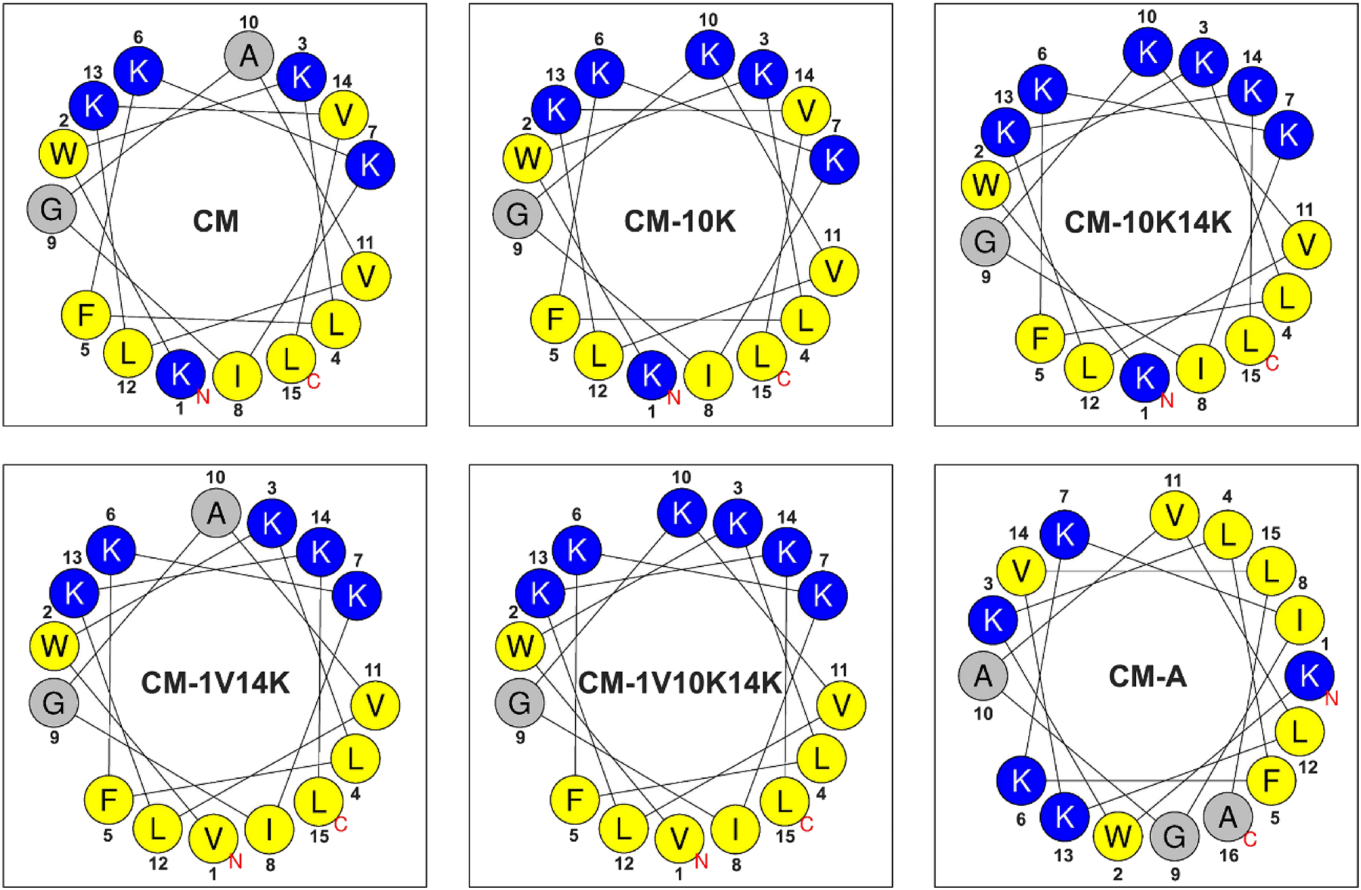
Helical wheel projections of CM derivatives obtained by HeliQuest. Positively charged and hydrophobic residues are shown in blue and yellow, respectively. Numbers represent the position of amino acid residues.

To increase the net positive charge, lysine was substituted in the CM parent peptide. CM-10K was modified by replacing alanine with lysine at position 10, while CM-10K14K was modified by replacing alanine at position 10 and valine at position 14 with two lysine, respectively, resulting in an improved hydrophilic face in helical wheel projection (Fig. 1). To increase the hydrophobicity, CM-1V10K14K was designed by substituting lysine with valine at position 1, where a lysine was embedded on the hydrophobic face in helical wheel projection (Fig. 1), thus improving the amphipathicity. CM-1V14K was created by retaining the original alanine at position 10 to investigate the effect of removing lysine under increasing hydrophobicity conditions. Furthermore, CM-A was designed by adding alanine at the end of the C-terminus where it links the hydrophobic and hydrophilic faces of the CM peptide, resulting in an improved secondary structure and increasing both the charge positivity and hydrophobicity.

The online Antimicrobial Peptide Calculator and Predictor (APD3 Server: https://aps.unmc.edu/prediction) was used to analyze the peptide sequences. The online programs I-TASSER (http://zhanglab.ccmb.med.umich.edu/I-TASSER/) and HeliQuest (https://heliquest.ipmc.cnrs.fr/) were exploited to determine three-dimensional structures and helical wheel projections, respectively.

### Peptide synthesis

All peptides were synthesized by China Peptides Co., Ltd. (Shanghai, China) with solid-phase methods using N-(9-fluorenyl) methoxycarbonyl (Fmoc) chemistry. They were amidated at the C-terminus and obtained as trifluoroacetate salts. The purity of peptides was confirmed to be higher than 95% using analytical reverse-phase high-performance liquid chromatography (RP-HPLC). The masses of these peptides were determined using electrospray ionization mass spectrometry (ESI–MS). Lyophilized peptides were dissolved in ultrapure (type 1) water at a concentration of 10 mg/mL and stored at − 20 °C until subsequent testing. For peptide dipping on catheters, lyophilized peptides were dissolved and used immediately.

### Bacterial strains and culture conditions

The *S. epidermidis* ATCC 35984 (biofilm-producer), *Staphylococcus aureus* ATCC 25923, *Enterococcus faecalis* ATCC 29219, *Acinetobacter baumannii* ATCC 19606, *Klebsiella pneumoniae* ATCC 27736 and *Pseudomonas aeruginosa* ATCC 27853 were grown overnight on trypticase soy agar (TSA; BD Bacto™), then subcultured in TSB and incubated at 37 °C for 16–18 h before each experiment. Stock bacterial cultures were kept frozen at − 80 °C in trypticase soy broth (TSB; BD Bacto™, USA) containing 20% glycerol.

### Antibacterial activity determination

The antimicrobial activity of peptides against *S. epidermidis* and five strains of both gram-positive and gram-negative bacteria frequently involved in catheter-related infections including *S. aureus*, *E. faecalis*, *A. baumannii*, *K. pneumoniae*, and *P. aeruginosa* was compared to that of vancomycin (Vancomycin hydrochloride, BioChemica, UK) using a modified version of the National Committee for Clinical Laboratory Standards (NCCLS) broth microdilution method^21^. Two-fold serially diluted (0.98–250 µg/mL) aliquots of the peptides were prepared in type 1 water and added to sterile 96-well polypropylene microplates (Nunclon Delta-Treated; Thermo Scientific™, Denmark) in a volume of 50 µL, followed by the addition of 50 µL of bacterial suspensions (2–7 × 10^5^ CFU/mL) in Mueller Hinton Broth (MHB; BD Difco™, USA). Uninoculated MHB and bacterial cultures without peptides were used as negative and positive controls, respectively. After culturing in an incubator shaker for 24 h at 37 °C, the lowest concentration at which the peptide inhibited bacterial growth as observed by visual inspection was taken as the minimal inhibitory concentration (MIC)^22^. Fifty microliter aliquots from each non-turbid well identified in the MIC experiment were plated on TSA for determination of minimal bactericidal concentration (MBC) by colony count assay^21^. MBC was defined as the lowest concentration of peptide that showed no bacterial growth on an agar plate. All experiments were performed in triplicate.

### Toxicity test

#### Hemolytic activity

The quantity of hemoglobin released by treatment was used as a measure of a peptide’s hemolytic activity against human red blood cells (hRBCs)^23^. Venous blood was collected from a healthy volunteer in plastic blood collection tubes with lithium heparin (BD Vacutainer™, USA) and centrifuged at 2000×*g* for 5 min to separate the plasma and buffy coat. The hRBCs obtained were washed at least three times and diluted to 4% (vol/vol) in phosphate-buffered saline (PBS; Caisson Laboratories, USA). The same volume of the hRBC suspension (50 µL) was incubated with twofold, serially diluted concentrations (0.98–250 μg/mL) of peptide dissolved in PBS for 1 h at 37 °C. The hRBCs incubated in 0.1% Triton X-100 and PBS were used as positive (100% hemolysis) and negative (0% hemolysis) controls, respectively. The release of hemoglobin into the supernatants was monitored by measurement of absorbance at 405 nm using a Multiskan SkyHigh microplate reader (Thermo Scientific™, Singapore). The percentage of hemolysis was calculated as previously described^24^.

Experiments involving human volunteers were in compliance with ethical standards and received approval from the Ethics Committee of Thammasat University (COA No. 066/2562). All participants voluntarily provided written informed consent.

#### Cytotoxicity

The cytotoxicity of the candidate peptide on L929 mouse fibroblast cell lines (NCTC clone 929) were evaluated using the 3-(4,5-dimethylthiazol-2-yl)-2,5-diphenyltetrazolium bromide (MTT) assay following the published protocol^25^. L929 cells (3–5 × 10^4^) in RPMI 1640 medium, supplemented with 10% (vol/vol) heat-inactivated fetal bovine serum, 2 mM l-glutamine, 0.2% (wt/vol) sodium bicarbonate, 100 U/mL penicillin and 100 mg/mL streptomycin were placed into each well of 96-well plates and incubated in 5% CO_2_ at 37 °C overnight. After removal of media, the two-fold serially diluted peptide was added to achieve final concentrations of 0.98–250 µg/mL. Plates were then incubated at 37 °C under 5% CO_2_ for 24 h. The RPMI 1640 medium with and without L929 seeded cells were used as positive and negative controls, respectively. After treatment, MTT (Sigma-Aldrich, USA) was added to a final concentration of 0.5 mg/mL and suspensions incubated for 4 h at 37 °C under 5% CO_2_. Supernatants from each well were then discarded, and 150 µl of dimethyl sulfoxide (DMSO; Fisher BioReagents™, UK) was added and gently mixed to dissolve the crystallized formazan. Absorbance was measured at 570 nm using a Multiskan SkyHigh microplate reader. Cell viability (%) was estimated according to the following formula:

$${\text{Cell}}\;{\text{viability}}\left( \% \right) = \left[ { \frac{{{\text{Abs}}_{{{\text{Treated}}}} - {\text{Abs}}_{{{\text{Blank}}}} }}{{{\text{Abs}}_{{{\text{Untreated}}}} - {\text{Abs}}_{{{\text{Blank}}}} }} } \right] \times 100$$

### Antibiofilm activity determination

#### Biofilm cell viability

The assessment of biofilm cell viability of CM-10K14K was conducted by the Calgary Biofilm Device (CBD)^26^ with some modifications. A suspension of *S. epidermidis* was exposed to serially diluted peptide concentrations ranging from 0.98 to 250 μg/mL within a 96-well microtiter plate covered with 96-peg lids (NuncTM Immuno TSP Lids, Denmark). After an incubation period of 24 h at 37 °C, the peg lids were rinsed to remove culture media and non-adherent cells. Following this, the peg lids underwent a washing process and were transferred onto new plate containing PBS. For the removal of biofilms from the peg lids, a water bath sonicator (Bandelin SONOREXTM, Germany) was employed. The detached biofilms were then serially diluted and plated on TSA. Following the incubation period, colonies were counted, and the biofilm cell viability was calculated.

#### Biofilm eradication

The effect of the peptide against established biofilm was evaluated following a previously described method with some modifications^26,27^. Bacterial suspension was added into 96-well microplates and were subsequently covered with 96-peg lids. After incubation at 37 °C for 24 h, the 96-peg lids underwent a rinsing step to eliminate culture media and non-adherent cells and the established grown biofilms adhering to the 96-peg lids were exposed to various concentration of peptide (ranging from 0.98 to 250 μg/mL) at 37 °C for an additional 24 h. Following this exposure period, the 96-peg lids were washed and transferred onto new plates containing PBS. Biofilm was removed from 96-peg lids by sonicator and the biofilms were serially diluted and cultured on TSA. After incubation at 37 °C for 24 h, the CFUs were counted, and the eradication of preformed biofilm by peptides was calculated according to the equation shown below:

$${\text{Biofilm}}\;{\text{eradication }}\left( \% \right) = \left( {\frac{{{\text{CFU}}_{{{\text{Untreated}}}} - {\text{CFU}}_{{{\text{Treated}}}} }}{{{\text{CFU}}_{{{\text{Untreated}}}} }}} \right) \times 100$$

### Biostability determination

Proteolysis of peptide by proteinase K was determined using the growth inhibition assay described by Jia et al.^28^ with some modifications. *Staphylococcus epidermidis* cell suspensions were prepared as described in MIC determination and incubated with peptide at final concentration ranging from 0.98 to 250 µg/ml in the presence of 0.01 mg/mL (final concentration) proteinase K at 37 °C with continuous shaking at 200 rpm. After 6 and 24 h of incubation, the growth inhibitory effect was determined by measuring the absorbance at 620 nm using a Multiskan SkyHigh microplate reader. The percentage of relative survival rate was calculated as follows:

$${\text{Relative}}\;{\text{survival}}\;{\text{rate}}\left( \% \right) = \left( {\frac{{{\text{Abs}}_{{{\text{Treated}}}} }}{{{\text{Abs}}_{{{\text{Untreated}}}} }}} \right) \times 100$$

where ${\text{Abs}}_{\text{Treated}}$ was absorbance value of *S. epidermidis* cells after incubation with peptide in the presence of proteinase K, ${\text{Abs}}_{\text{Untreated}}$ was that of *S. epidermidis* cells incubated with ultrapure water in the presence of proteinase K.

### Circular dichroism (CD) analysis

The secondary structure of peptide induced by membrane-mimetic surroundings was evaluated on a Jasco-815 spectropolarimeter (Jasco, Japan) at 25 °C, using a 0.1-cm-path-length rectangular quartz cell^29^. The peptides were dissolved at a final concentration of 0.2 mg/mL in PBS, 30 mM SDS surfactant (Sigma-Aldrich, Japan) or 50% (vol/vol) TFE (Merck, USA) to mimic an aqueous environment, negatively charged bacterial membranes, and hydrophobic parts of microbial membranes, respectively, and conformational changes assessed. Their spectra were recorded in the 190–250 nm range at a scanning speed of 10 nm/min with at least three replicates for each condition. The CD signal spectra were then transformed to mean residue ellipticity using the following equation:

$$\uptheta _{{\text{M}}} = \left( {\frac{{\theta_{obs} }}{10}} \right) \times \left( {\frac{{{\text{M}}_{{{\text{RW}}}} }}{{{\text{c}} \cdot 1}}} \right)$$

where θ_M_ is residue ellipticity (deg. M^−1^ m^−1^), θ_obs_ is detected ellipticity adjusted for buffer at a given wavelength (mdeg), M_RW_ is residue molecular weight (M_W_/number of amino acids), c is peptide concentration (mg/mL), and l is path length (cm).

### Time-kill kinetics assay

Time-kill curve analyses were assessed by culturing the mid-logarithmic growth phase of *S. epidermidis* in MHB medium^24^, in the presence of peptide at MIC or MBC concentrations. After incubation at 37 °C, ten microliters of bacterial suspensions were aliquoted at various time intervals (0.25, 0.5, 1, 2, 4, 6, 8 and 24 h). The aliquots were ten-fold serially diluted in MHB, and 100 µL of each dilution was plated onto TSA. After 24 h of incubation at 37 °C, the number of CFU was determined.

### Mode of action

#### Flow cytometry analysis

Flow cytometry was employed to investigate membrane-lytic mechanisms induced by peptide. Propidium iodide (PI, Sigma-Aldrich, USA), a DNA intercalating dye, was employed to assess the bacterial membrane permeability, while bis-(1,3-dibutylbarbituric acid) trimethine oxonol (BOX; Sigma-Aldrich, USA) was used to monitor changes in the bacterial membrane potential^6^. Cultured *S. epidermidis* was washed and the concentration adjusted to an absorbance reading of 0.05 at 620 nm in 1 × PBS. The bacterial suspensions were treated with 1 × MIC of the peptide candidate, then incubated at 37 °C for 5, 15 or 30 min with constant shaking at 140 rpm. The negative control consisted of untreated bacterial cells; the positive control of bacterial cells heated at 70 °C for 30 min. After treatment, bacterial cells were collected by centrifugation at 10,000×*g* for 10 min and washed with 1 × PBS to remove unbound peptide before adding fluorescent probes. PI and BOX were added to each cell suspension to final concentrations of 1 µg/mL and 0.0625 µM, respectively, for 15 min in the dark before analysis. A flow cytometer (CytoFlex, Beckman Coulter, USA) was used to measure the fluorescent signals at a laser excitation wavelength of 488 nm. Forward scatter (FSC), side scatter (SSC), red fluorescence (585/42 nm) generated by PI and green fluorescence (530/30 nm) from BOX were each collected. 25,000 cells were counted in each sample in accordance with their scatter parameters and the data were investigated using logarithmic scales and Kaluza software version 2.1 (Beckman Coulter, USA).

#### Scanning electron microscopy

Under scanning electron microscopy (SEM), the surface morphological and biofilm formation of peptide-treated bacteria were evaluated. *Staphylococcus epidermidis* cultures were washed and adjusted to an OD_620_ of 0.05 in 300 mL of 1 × PBS. Bacterial suspensions were then incubated with peptide at a final concentration of 1 × MIC at 37 °C for 15 min. After treatment, cells were harvested by centrifugation at 10,000×*g* for 20 min and washed with 1 × PBS to remove the unbound peptide. As described previously^25^, the treated bacterial cells were fixed in 2.5% (vol/vol) glutaraldehyde at 4 °C for 12 h. The samples were then washed twice with PBS and dehydrated for 15 min in each of a graded ethanol series (50%, 70%, 90% and 100%), followed by transfer to a mixture (1:1) of ethanol and tertiary butanol for 20 min, and then to pure tertiary butanol for 20 min. After lyophilization and gold coating, the samples were analyzed using a scanning electron microscope (Hitachi SU8020, Tokyo, Japan).

#### Transmission electron microscopy

Alterations in structural integrity and membrane destruction of bacteria were further observed under transmission electron microscopy (TEM). Bacterial cells were prepared and incubated with peptide as previously described^25^. After harvesting the bacterial pellets, samples were fixed overnight in glutaraldehyde at a concentration of 2.5% (vol/vol). Afterward, pellets were washed twice with 1 × PBS and post-fixed for 2 h with 1% osmium tetroxide in 1 × PBS. The fixed samples were then washed twice with 1 × PBS, followed by dehydration for 15 min each in a graded ethanol series (50%, 70%, 90%, and 100%). After transfer to a mixture of absolute acetone (1:1) and epoxy resin (1:3) for 1 h, samples were placed in absolute acetone for 20 min, followed by transfer to pure epoxy resin and held for 12 h. A glass knife (ultramicrotome) was used to cut ultrathin slices. After post-staining with lead citrate and uranyl acetate, these slices were examined using a transmission electron microscope (Hitachi HT7700, Japan).

#### DNA-binding assay

A gel retardation assay was employed to investigate the interaction of peptide candidate with intracellular targets such as DNA^30^. Overnight *S. epidermidis* cultures were harvested by centrifugation at 4000×*g* for 10 min and genomic DNA was extracted using the E.Z.N.A.® Bacterial DNA kit (Omega Bio-Tek, USA). Quantity and purity of the DNA were determined using NanoDrop™ One Microvolume UV–Vis Spectrophotometers (Thermo Scientific™, USA). The serially diluted concentrations (0.98–250 μg/mL) of candidate peptides were suspended in binding buffer [50 µg/mL bovine serum albumin (BSA), 20 mM KCl, 10 mM Tris–HCl (pH 8.0), 1 mM ethylene diamine tetraacetic acid (EDTA), 1 mM dithiothreitol (DTT) and 5% glycerol and then incubated with 143.5 ng of genomic DNA at 37 °C for 1 h. Subsequently, the samples were mixed with 2 µL of 6× loading dye (Vivantis, Malaysia) and 10-μL aliquots were added to 1% agarose gel electrophoresis in 1 × Tris borate-EDTA buffer (89 mM Tris base, 89 mM boric acid and 2 mM EDTA, pH 8.0; Vivantis, Malaysia).

### Assessment of prevention of biofilm formation by CM-10K14K-impregnated catheters

#### Peptide dip-coating of catheters by physical adsorption

**Figure 2:**
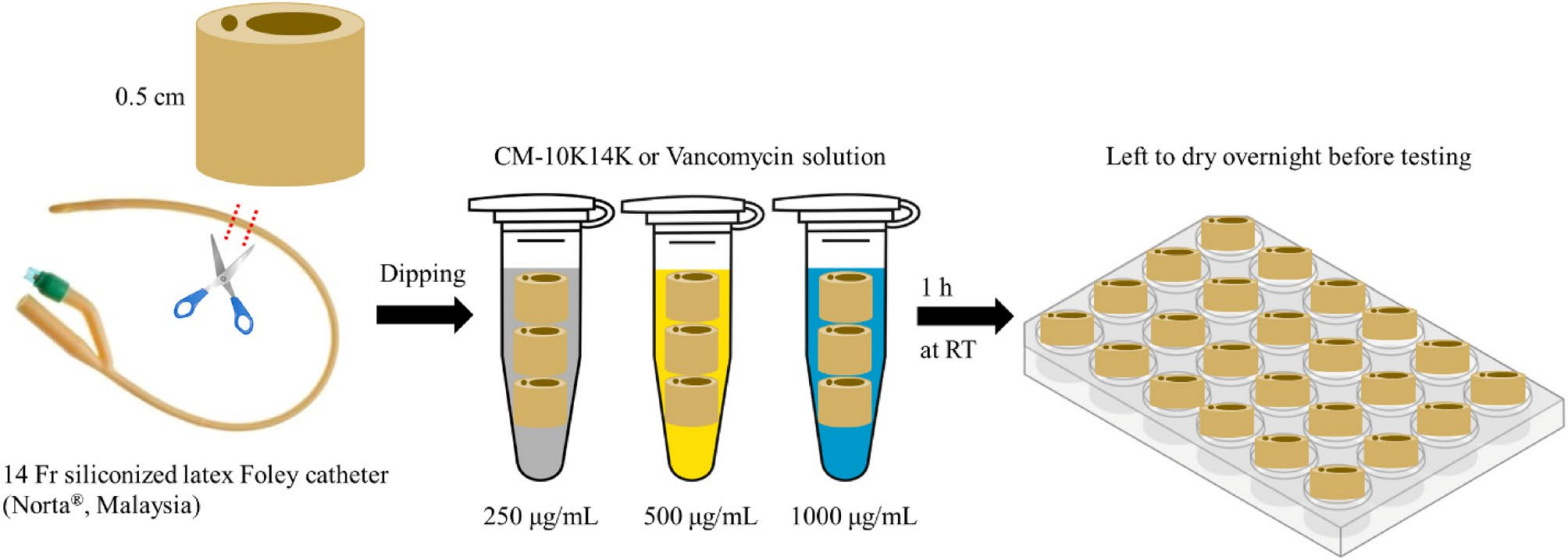
Workflow of peptide dip-coating on catheters.

A sterilized 14 Fr siliconized latex Foley catheter (Norta®, Malaysia) was cut into 0.5 cm sections and dipped into one of three concentrations (250, 500 and 1000 µg/mL) of CM-10K14K, or into vancomycin freshly prepared in ultrapure water for 1 h at room temperature (Fig. 2), a modified protocol^31^. The dipped segments were placed in wells of a 24-well plate (1 section per well) and left to dry overnight before testing.

#### In vitro catheter-associated infection model with biofilm-forming *S. epidermidis*

The peptide-impregnated catheter segments were placed in the wells of sterile 24-well tissue culture plates (Corning, USA) and 1 mL of TSB containing approximately 10^7^ CFU/mL of *S. epidermidis* was added to each well (3 catheter segments per concentration). The plates were then incubated at 37 °C for 6 and 24 h. After incubation, the treated segments were transferred to new 24-well plates for future determination of biofilm biomass. Supernatants were collected to determine if viable cells remained; each well containing biofilm, along with its corresponding control media, was fixed and stained with 0.1% CV as described in Supplementary Information (Sect. 1). The percentage of biofilm formation compared to controls was calculated.

#### Determination of viable planktonic cells by colony count assay

The supernatants were ten-fold serially diluted in 0.85% NaCl, and 10 µL of each dilution (n = 3) was dropped onto TSA. After 24 h of incubation at 37 °C, the CFUs were counted.

#### Assessment of biofilm biomass on peptide-impregnated catheters by crystal violet staining

The treated catheter segments were washed twice in 0.85% NaCl without disturbing the biofilm, then transferred to tubes containing 1 mL of 0.1% CV stain for 15 min. The stained segments were gently washed, until the supernatants of the negative controls (untreated segments) were clear and left to dry for 1 h. The segments were transferred to tubes containing 600 µL of 95% ethanol for solubilization, followed by sonication for 10 min and vertexing for 30 s three times. The Multiskan SkyHigh microplate reader was used to measure the absorbance at 570 nm.

### Statistical Analysis

All experiments were carried out in triplicate and results are presented as mean ± standard deviation (SD). The data were analyzed with GraphPad Prism (version 9.0), using a two-way ANOVA followed by the Tukey's post hoc test. *P* values of < 0.05 were considered significant.

## Results

### The designed peptides showed potent antibacterial activity

The newly designed peptides derived from C(1–7)M(2–9) were tested against the biofilm-forming strain of *S. epidermidis* (Table 1). Among these peptides, the most potent antibacterial activity was demonstrated by the lysine-substituted derivatives with increased net charge, CM-10K and CM-10K14K with MICs (3.91 µg/mL) and MBCs (7.81 µg/mL), comparable to those of vancomycin, a conventional antibiotic. Derivatives with increased hydrophobicity (CM-1V10K14K, CM-1V14K and CM-A) had two-fold higher MBC values than that of the parental peptide.

**Table 1:** Amino acid sequence, key physicochemical parameters, antibacterial, and hemolytic activity of designed peptides against *Staphylococcus epidermidis*.

Peptide	Amino acid sequence	MW^a^	aa^b^	Net charge	Pho%^c^	µH^d^	MIC (MBC)^e^ (µg/mL)	HC_50f._ (µg/mL)	TI^g^
Parent peptide									
C(1–7)M(2–9)	KWKLFKKIGAVLKVL-NH_2_	1770.34	15	+ 5	60	0.483	7.81 (7.81)	88.89 ± 4.62	11.38
Derivatives with increased positive charge									
CM-10K	KWKLFKKIGKVLKVL-NH_2_	1827.44	15	+ 6	53	0.568	3.91 (7.81)	85.78 ± 9.01	21.94
CM-10K14K	KWKLFKKIGKVLKKL-NH_2_	1856.48	15	+ 7	46	0.671	3.91 (7.81)	561.90 ± 88.19	143.71
Derivatives with increased hydrophobicity									
CM-1V10K14K	VWKLFKKIGKVLKKL-NH_2_	1827.44	15	+ 6	53	0.819	3.91 (15.63)	118.90 ± 8.19	30.41
CM-1V14K	VWKLFKKIGAVLKKL-NH_2_	1770.34	15	+ 5	60	0.732	7.81 (15.63)	131.70 ± 9.23	16.86
CM-A	KWKLFKKIGAVLKVLA-NH_2_	1841.42	16	+ 6	62	0.459	7.81 (15.63)	42.58 ± 1.98	5.45
Conventional antibiotic (Control)									
Vancomycin	–	1449.25	–	–	–	–	3.91 (7.81)	–	–

^a^MW, molecular weight (g/mol) measured by mass spectroscopy (MS).

^b^aa, number of amino acids.

^c^Pho%, the percentage of hydrophobic residues.

^d^µH, the mean hydrophobic moment determined using website: http://heliquest.ipmc.cnrs.fr/.

^e^MIC (MBC), minimum inhibitory concentration (minimum bactericidal concentration).

^f^HC_50_, the hemolytic concentration of peptide that causes 50% hemolysis relative to the positive (Triton-X) control.

^f^TI, therapeutic index defined as the ratio of the HC_50_ value to the MIC value.

### CM-10K14K exhibited potent antimicrobial activity with low toxicity

**Figure 3:**
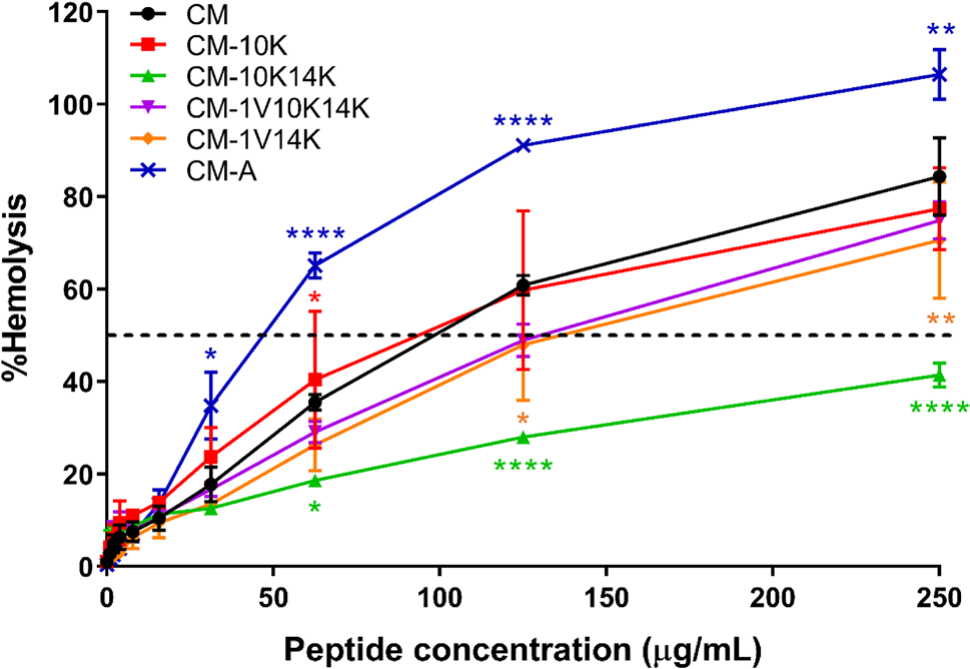
Hemolytic activity of peptide derivatives against human red blood cells. The experiments were performed in triplicate and the data are expressed as the mean ± SD. *P* values were determined using a two-way ANOVA with Tukey’s post hoc test. Significant differences are indicated in GP style: > 0.05 (ns), ≤ 0.05 (*), ≤ 0.01 (**), ≤ 0.001 (***), ≤ 0.0001 (****). The horizontal dashed line represented 50% hemolysis.

The potential toxicity of the peptides was evaluated by their ability to lyse hRBCs and their toxicity against L929 mouse fibroblast cells. The lysis effect of peptides on hRBCs is shown in Fig. 3; HC_50_ was defined as the concentration of peptide causing 50% lysis of hRBCs (Table 1)^32^. The results showed that all derivatives, except CM-10K (with less hydrophobicity and higher mean hydrophobic moment than the parental peptide) had improved selectivity as demonstrated by higher HC_50_ values. Of all peptides, CM-A (an alanine-substituted peptide with the highest hydrophobicity and the lowest mean hydrophobic moment) displayed the strongest hemolytic activity with an HC_50_ of 42.58 ± 1.98 µg/mL. In contrast, CM-10K14K (a lysine-enriched peptide) showed the least hemolytic activity based on having the highest HC_50_ (561.90 ± 88.19 µg/mL). At both the MIC and MBC of CM-10K14K, its hemolytic activity was less than 9%.

**Figure 4:**
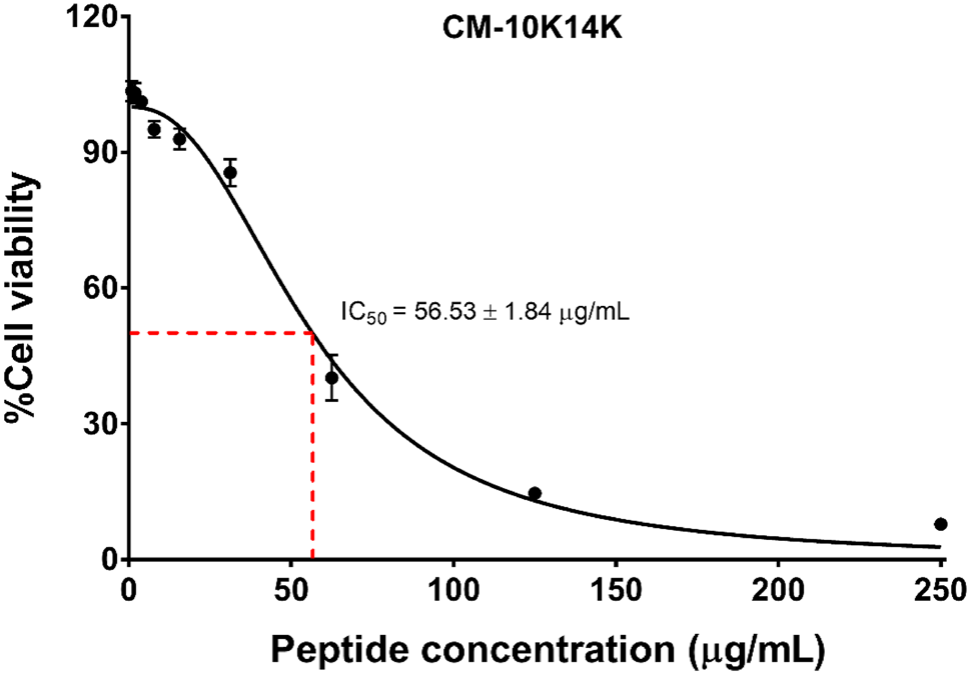
The half maximal inhibitory concentration (IC_50_) of CM-10K14K on L929 mouse fibroblast cells at 24 h. The experiments were performed in triplicate and the data were analyzed by GraphPad Prism 7 Software and are expressed as the mean ± SD. The dashed lines represented the determination of the IC_50_ value.

Given the low degree of hemolytic activity of CM-10K14K, its cytotoxicity was further assessed using L929 mouse fibroblast cells (Fig. 4). The toxicity on these cells was also low, with an IC_50_ of 56.53 ± 1.84 µg/mL. After peptide treatment, the cell viability was 100% and 92.87 ± 1.82 µg/mL at the MIC and MBC, respectively. Based on its antibacterial and toxicity profile, peptide CM-10K14K was selected for further analysis. CM-10K14K, a candidate peptide, also showed broad-spectrum antibacterial activity against a wide range of gram-positive and gram-negative bacteria were frequently involved in catheter-related infections with MICs ranging from 7.81 to 31.25 µg/mL and MBCs ranging from 7.81 to 62.5 µg/mL as shown in Table 2. Among tested strains, CM-10K14K displayed the strongest antibacterial activity against *S. epidermidis* with the lowest MIC and MBC values.

**Table 2:** Minimum inhibitory concentration (MIC) and minimum bactericidal concentration (MBC) of CM-10K14K against bacteria frequently involved in catheter-related infections^60–62^.

Bacterial strains	MIC (µg/mL)	MBC (µg/mL)
Gram-positive bacteria		
*Staphylococcus aureus* ATCC 25923	7.81	7.81
*Enterococcus faecalis* ATCC 29219	31.25	31.25
Gram-negative bacteria		
*Acinetobacter baumannii* ATCC 19606	7.81	15.63
*Klebsiella pneumoniae* ATCC 27736	15.63	31.25
*Pseudomonas aeruginosa* ATCC 27853	15.63	62.5

### CM-10K14K inhibited and eradicated *S. epidermidis* biofilm

**Figure 5:**
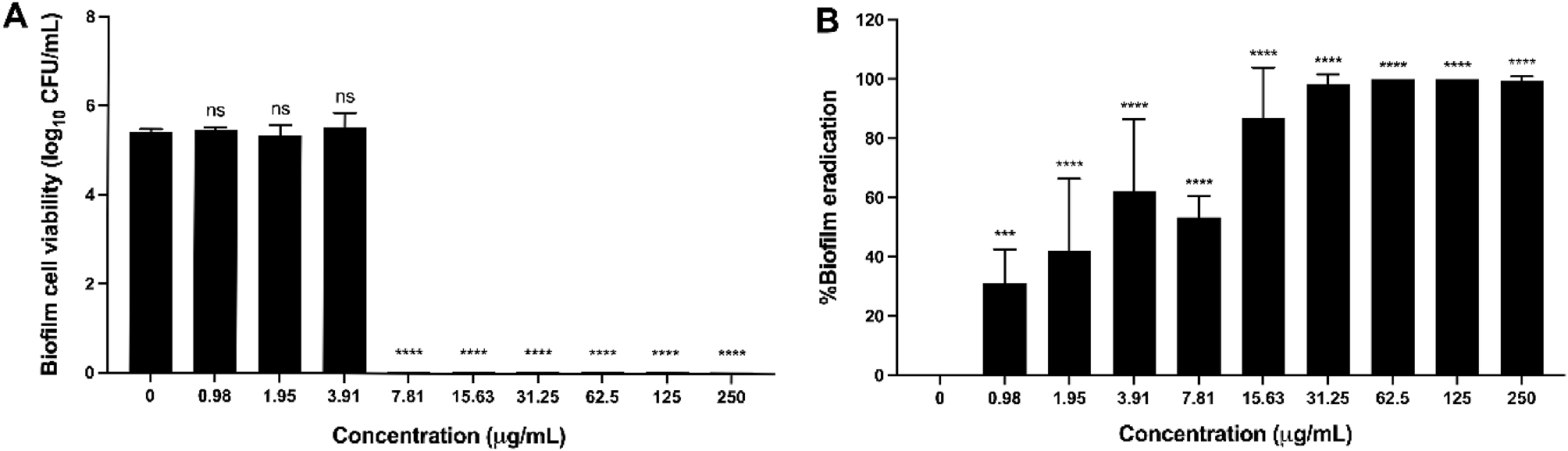
Biofilm cells viability (A) and Biofilm eradication activity (B) of *S. epidermidis* ATCC 35,984 after treated with different concentration of CM-10K14K. The experiments were performed in triplicate and the data were expressed as the mean ± SD. *P* values were determined using a two-way ANOVA with Tukey’s post hoc test. Significant differences are indicated in GP style: > 0.05 (ns), ≤ 0.05 (*), ≤ 0.01 (**), ≤ 0.001 (***), ≤ 0.0001 (****).

At a concentration of 7.81 µg/mL (MBC) and higher, CM-10K14K killed all *S. epidermidis* cells grown within the biofilm indicating the bactericidal effect of this peptide and consequently the prevention of biofilm formation (Fig. 5A). These findings are consistent with the reduction in biofilm biomass observed through crystal violet staining (Supplementary Fig. 1). Notably, at a concentration that inhibits the bacterial growth or MIC (3.91 µg/mL), *S. epidermidis* biofilm cells remained viable. This observation highlights a significant difference in the response of planktonic and biofilm cells to the peptide treatment. In addition to biofilm formation inhibitory activity, CM-10K14K displayed a dose-dependent eradication activity on mature or established *S. epidermidis* biofilm. More than 90% of mature biofilm was eradicated at a peptide concentration equal to or more than 31.25 μg/ml as shown in Fig. 5B.

### CM-10K14K could inhibit the bacterial growth in the presence of protease enzyme

**Figure 6:**
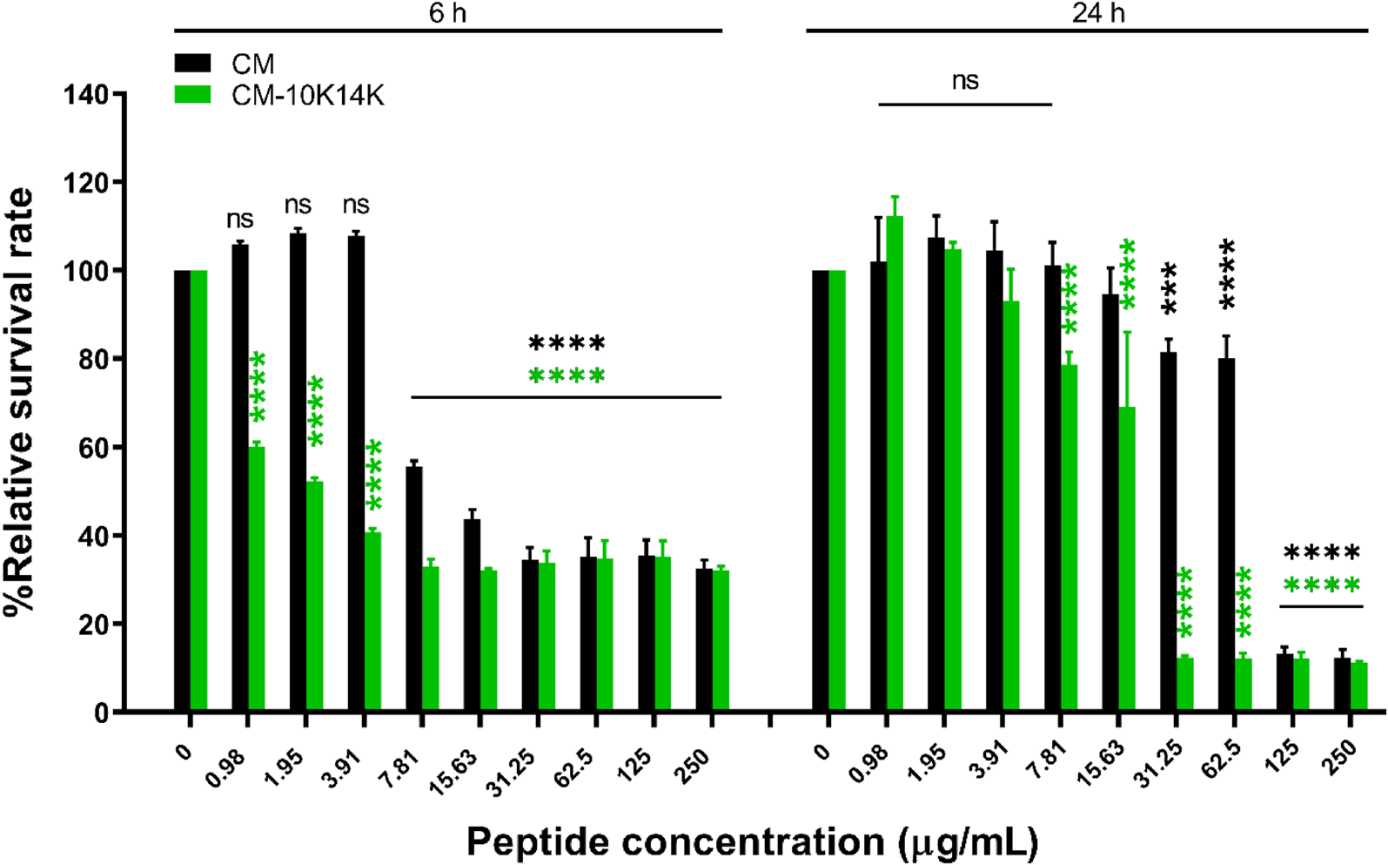
The relative survival rate of *S. epidermidis* ATCC 35984 after treated with CM and CM-10K14K in the presence of proteinase K for 6 and 24 h. The experiments were performed in triplicate and the data were expressed as the mean ± SD. *P* values were determined using a two-way ANOVA with Tukey’s post hoc test. Significant differences are indicated in GP style: > 0.05 (ns), ≤ 0.05 (*), ≤ 0.01 (**), ≤ 0.001 (***), ≤ 0.0001 (****).

Most AMPs are susceptible to protease degradation because their amino acid sequence served as excellent substrates for proteolytic cleavage. The biostability of CM-10K14K compared with CM parent peptide was determined in the presence of proteinase K, a broad-spectrum protease. CM-10K14K demonstrated higher tolerance to proteinase K compared to that of CM, as indicated by a reduced survival rate, as illustrated in Fig. 6. After 6 h of incubation, CM-10K14K demonstrated the ability to inhibit bacterial growth ranging from the lowest (0.98 µg/mL) to the highest tested concentration, while at least its MIC was needed for the CM peptide to kill the bacteria. After 24-h incubation, CM-10K14K at 31.25 and 62.5 µg/mL exhibited strong antibacterial activity, but the original CM lost its ability to kill bacteria. The growth inhibition results from the in vitro experiment were consistent with the potential cleavage site predicted by PeptideCutter (https://web.expasy.org/peptide_cutter/). The CM sequence exhibited 9 cleavage site positions, whereas the CM-10K14K sequence had 7 cleavage sites as shown in Supplementary Table 1.

### CM-10K14K adopted an α-helical structure in membrane-mimetic environments

**Figure 7:**
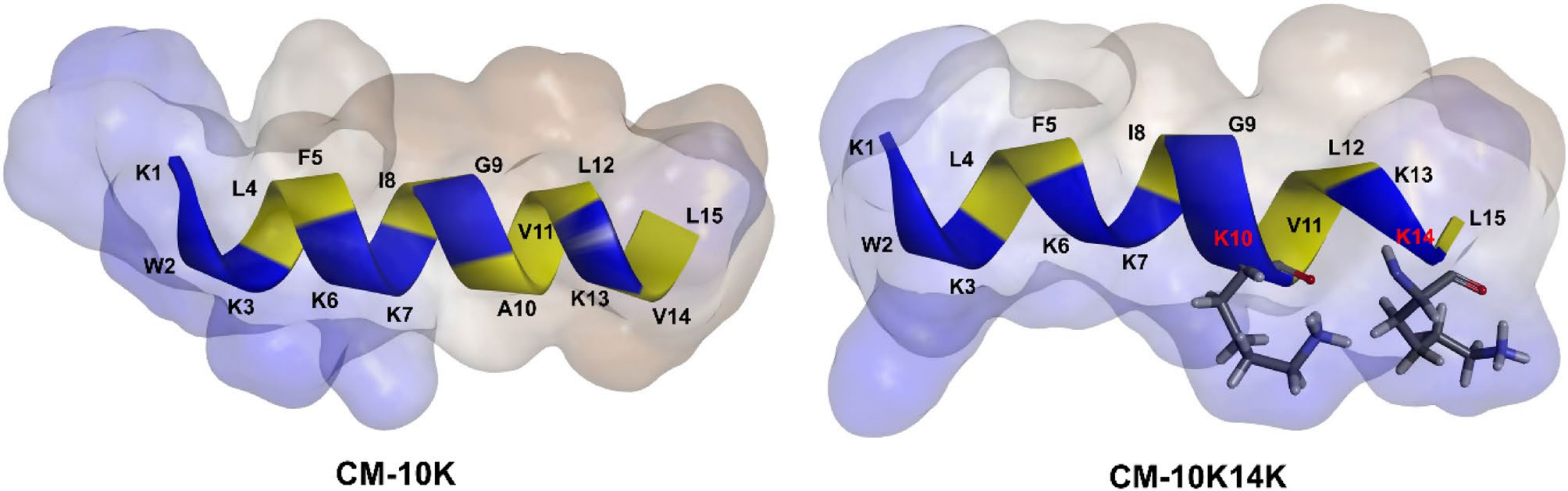
In silico three-dimensional (3D) molecular modeling, obtained by I-TASSER. Positively charged and hydrophobic residues are shown in blue and yellow, respectively. Numbers represent the position of amino acid residues.

**Figure 8:**
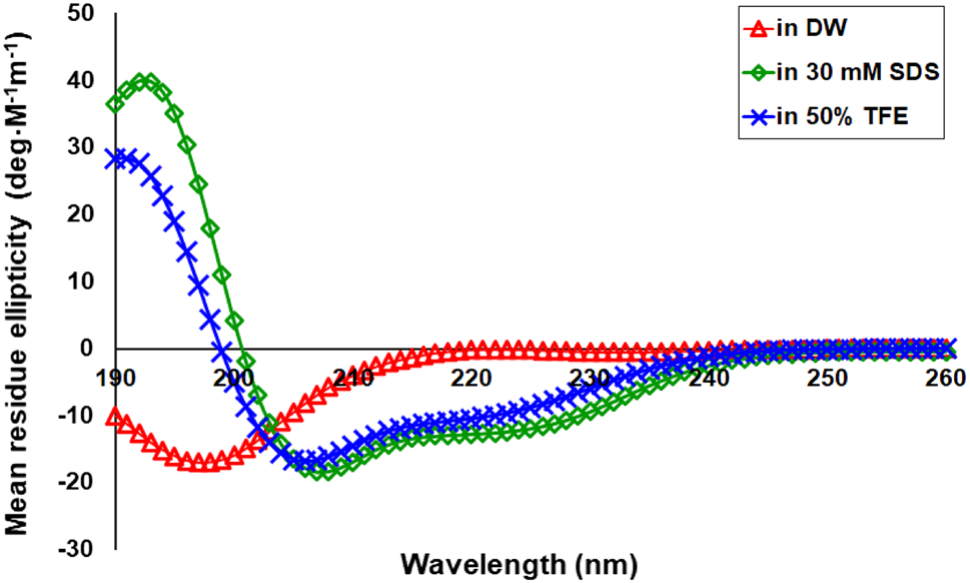
Circular dichroism spectra of CM-10K14K in aqueous solution (red), 30 mM SDS micelles (green) and 50% TFE (blue). The samples were loaded in a rectangular quartz cell (0.1 cm path length); the CD signals were measured at 25 °C using a Jasco-815 spectropolarimeter and recorded at a scanning speed of 10 nm/min in the wavelength range of 190–260 nm.

As suggested by I-TASSER and in silico molecular three-dimensional (3D) models using BIOVIA Discovery Studio Visualizer 2020, CM-10K14K and other peptides showed an α-helical structure (Fig. 7). The secondary structures adopted by peptides in membrane-mimic environments were further evaluated using CD spectroscopy (Fig. 8). In aqueous solution, the existence of a significant minimum peak of mean residue ellipticity at 198 nm suggested that CM-10K14K constructed random coil structures. In both 30 mM SDS and 50% TFE solutions, CM-10K14K revealed two negative bands at 222 nm and at 208 nm, and a positive band at 193 nm, characteristics of α-helical structures. These findings indicated that CM-10K14K formed an α-helical structure in membrane-mimicking conditions.

### CM-10K14K rapidly kills *S. epidermidis*

**Figure 9:**
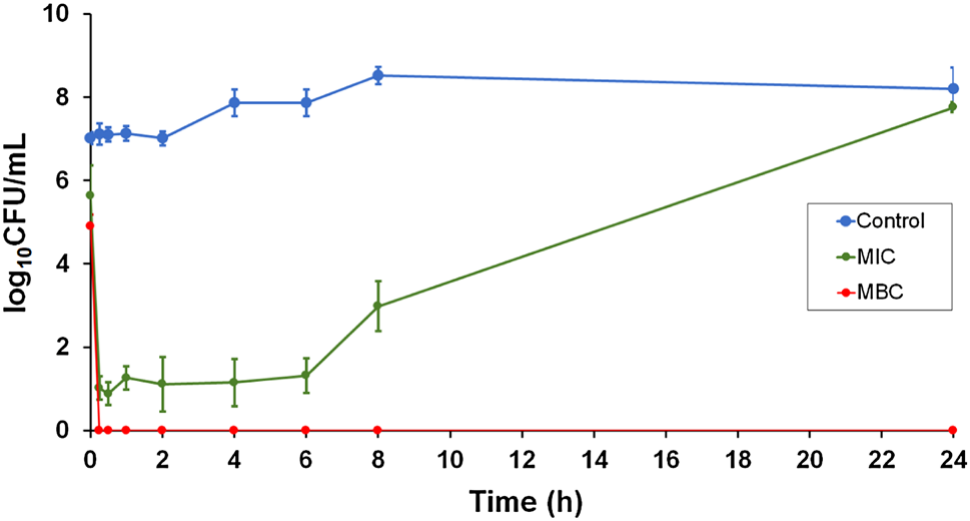
Bacterial-killing kinetics of CM-10K14K against *S. epidermidis* at MIC and MBC concentrations for 24 h post-treatment.

The killing kinetics of CM-10K14K against *S. epidermidis* were determined at peptide concentrations of 3.91 μg/mL (MIC) and 7.81 μg/mL (MBC) and compared to those of untreated bacteria (control). As shown in Fig. 9, CM-10K14K at MIC showed a strong inhibitory effect that reduced the concentration of viable *S. epidermidis* approximately 10^6^ CFU/mL within 15 min, and persisted at least 6 h after treatment. Beyond that time bacterial regrowth was observed. However, CM-10K14K at MBC totally eradicated bacteria within 15 min and no regrowth was observed through 24 h. Therefore, CM-10K14K displayed bactericidal activity which was rapid, and both concentration- and time-dependent.

### CM-10K14K disrupted bacterial membranes by depolarization and permeabilization

**Figure 10:**
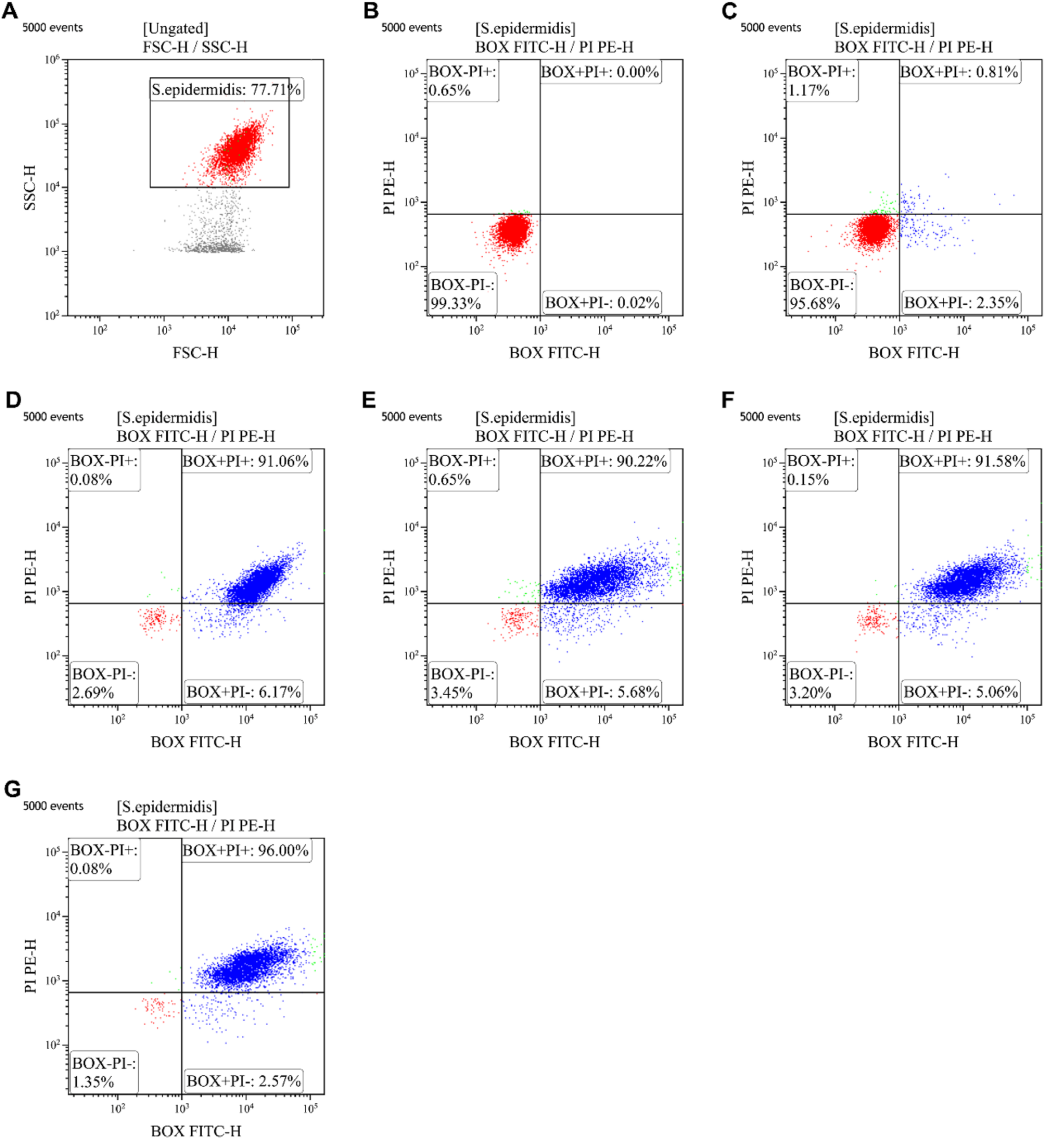
Flow cytometry analysis of *S. epidermidis* treated with CM-10K14K. (**A**) The tested bacterial population. (**B**) Untreated *S. epidermidis* without PI and BOX. (**C**) Untreated *S. epidermidis* with PI and BOX stained. The effect on membrane permeability (PI) and membrane depolarization (BOX) of *S. epidermidis* after treatment with 70 °C for 30 min (**D**), and CM-10K14K at 1 × MIC for 5, 15 and 30 min (**E**–**G**). The percentage of cell populations that fell in each gate are shown in the four corners of each plot. The experiments were performed in triplicate.

The mechanisms of antibacterial action of CM-10K14K were investigated using flow cytometry to determine if they were membrane-related. The positive fluorescent signals emitted by PI staining indicated that bacterial membrane integrity was severely compromised or pores had formed, whereas the fluorescent signals from BOX identified the depolarized bacterial cells. In flow cytometric analysis, FSC and SSC gating was used to define the bacteria for study (77.95 ± 0.81%; Fig. 10A). Unstained cells are used to set the negative population (Fig. 10B). Most untreated cells demonstrated negative fluorescent signals (95.34 ± 0.18%) indicating healthy and intact cell membranes (Fig. 10C). Heat treatment of bacterial cells at 70 °C for 30 min, a condition known to kill *S. epidermidis* within biofilms^33^, was utilized as a positive control in the experiment^34^. Heated bacterial cells had high levels (91.51 ± 0.32%) of depolarization and permeabilization (Fig. 10D). The membrane-active mechanism was investigated in a short-time course at the early phase of treatment, based on the rapid bactericidal activity of CM-10K14K observed in the killing kinetics experiments. The results showed that CM-10K14K at 1 × MIC induced time-dependent depolarization and permeabilization of 90.80 ± 0.51%, 91.36 ± 0.74% and 95.71 ± 0.26% of treated bacteria at 5, 15, and 30 min, respectively (Fig. 10E–G). These findings showed that CM-10K14K damaged *S. epidermidis* membranes in a time-dependent manner through depolarization and permeabilization, and supported their rapidity in the killing kinetics experiment.

### CM-10K14K inhibited biofilm formation and altered bacterial membrane structures

**Figure 11:**
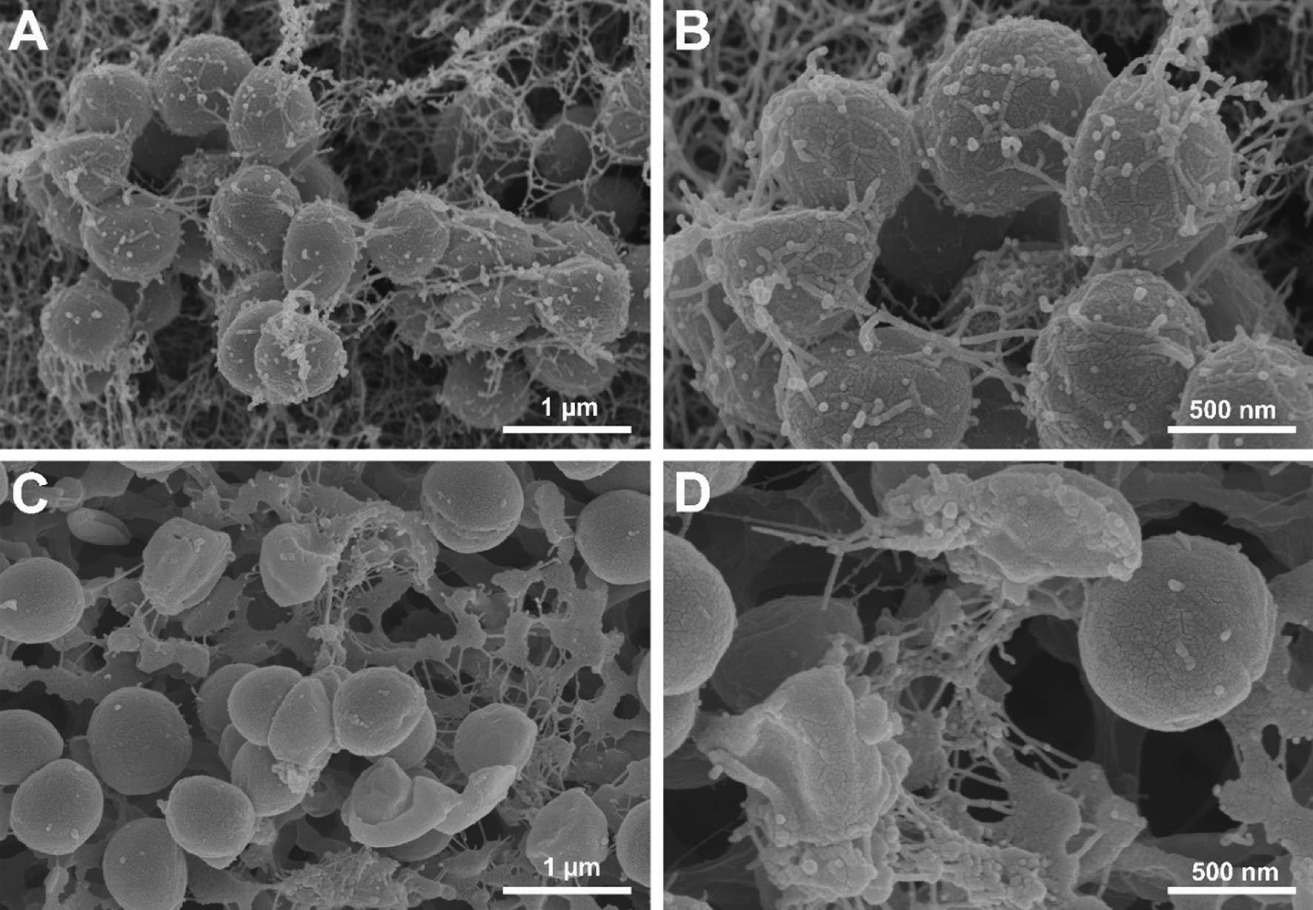
Scanning electron microscopic images of *S. epidermidis* treated with CM-10K14K. Untreated bacterial cells served as controls (**A**,**B**). Cells treated with 1 × MIC of CM-10K14K for 15 min are shown in (**C**,**D**).

Alterations in bacterial morphology and biofilm formation after peptide treatment were evaluated using SEM. Treatment with CM-10K14K at 1 × MIC for 15 min resulted in significant membrane corrugation, distortion and damage of *S. epidermidis* (Fig. 11C,D), as well as a reduction of extracellular matrix or biofilm when compared with controls. In contrast, the untreated bacterial cells showed more structural and adherent characteristics and became embedded within a biofilm matrix (Fig. 11A,B).

**Figure 12:**
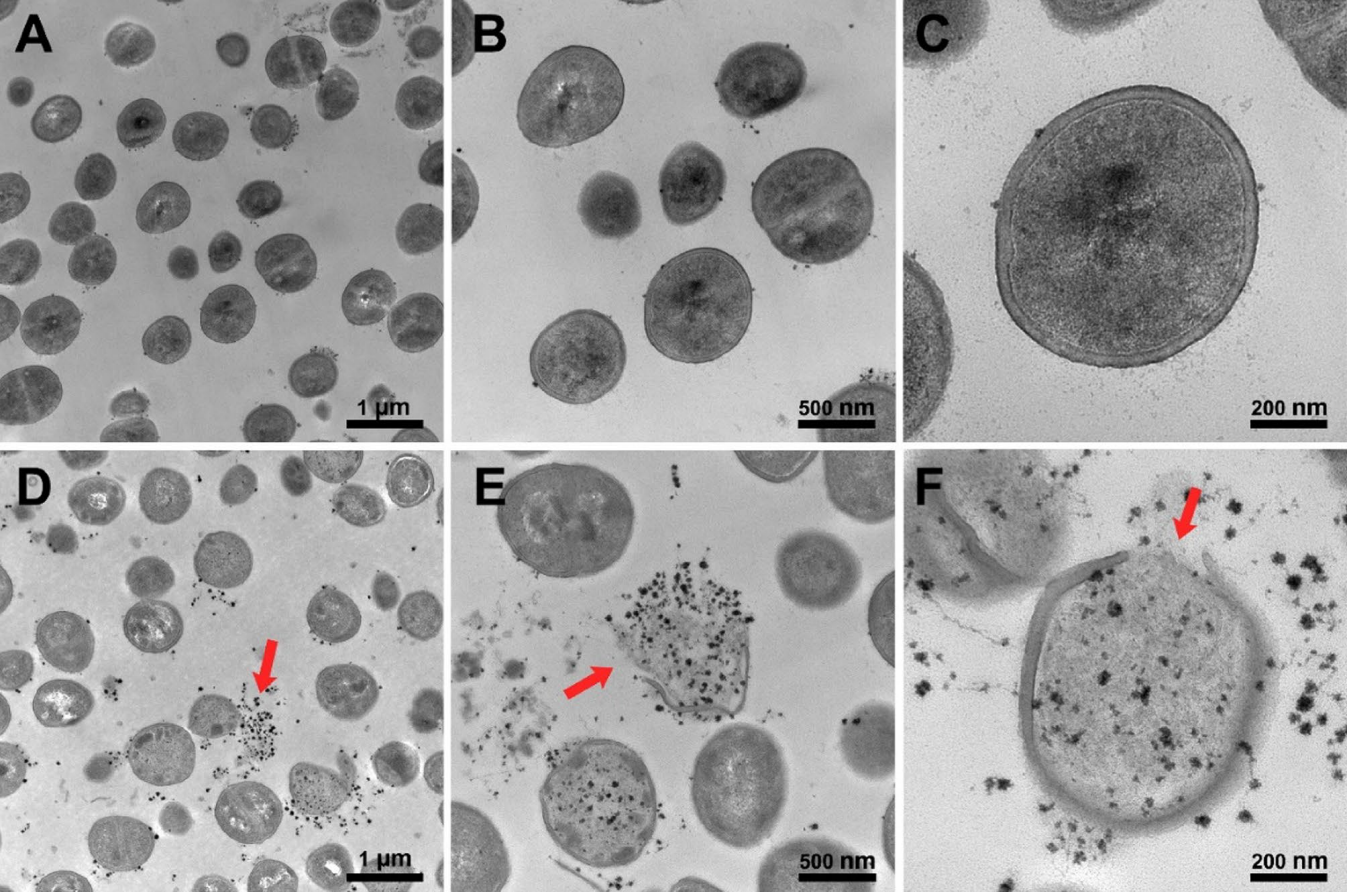
Transmission electron microscopic images of *S. epidermidis* treated with CM-10K14K. Untreated bacterial cells (**A**–**C**); cells treated with 1 × MIC of CM-10K14K for 15 min (**D**–**F**). The red arrows indicate cell membrane disintegration and dispersion of intracellular contents.

Cell structures at the nanometer scale and intracellular alterations in peptide-treated bacterial cells were investigated by TEM. Untreated bacterial cells were spherical in shape and had smooth cell walls (Fig. 12A–C). In contrast, cells treated with CM-10K14K were structureless, containing empty electron-transparent parts and amorphous clumps of various electron densities (Fig. 12D–F) as described previously^35^. The red arrows in Fig. 12 indicate cell membrane disintegration and dispersion of intracellular contents after exposure to CM-10K14K at MIC for 15 min. These TEM results agreed with the results from flow cytometry analysis, suggesting that CM-10K14K acted on bacterial membranes as a mechanism of its antibacterial activity.

### CM-10K14K interacts with *S. epidermidis* chromosomal DNA

**Figure 13:**
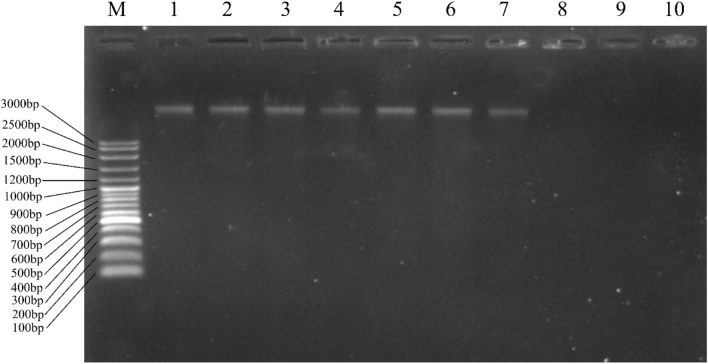
Analysis of the bacterial DNA-binding activity of CM-10K14K using a gel retardation assay. Concentrations of CM-10K14K, ranging from 0.98, 1.95, 3.91, 7.81, 15.63, 31.25, 62.5, 125 to 250 μg/mL (Lanes 2–10, respectively), were mixed with 143.5 ng DNA of *S. epidermidis*. Lane M and 1 represent the VC 100 bp Plus DNA Ladder (Vivantis, Malaysia) and genomic DNA without peptide treatment, respectively. The original gel is presented in Supplementary Fig. 2.

In addition to disrupting the bacterial membrane, some AMPs kill bacteria by penetrating the cells and interacting with intracellular components^36^. The DNA-binding ability of CM-10K14K was thus evaluated in a gel retardation assay. Twofold serial concentrations of peptide (0.98–250 µg/mL) were mixed with a fixed amount (143.5 ng) of genomic DNA extracted from *S. epidermidis.* The complexes were electrophoresed on a 1% agarose gel. When mixed with lower concentrations (0.98–31.25 µg/mL) of peptide, the genomic DNA was able to migrate through the gel (lanes 2–7) in the same manner as non-complexed DNA (lane 1). When the amount of peptide was increased (62.5–250 µg/mL), the genomic DNA was significantly retarded in the gel, indicating that it had become aggregated by CM-10K14K (Fig. 13). This finding suggested that CM-10K14K can bind to bacterial genomic DNA, which could be the intracellular target for bacterial killing mechanisms.

### CM-10K14K impregnated in catheters inhibits biofilm formation by *S. epidermidis*

**Figure 14:**
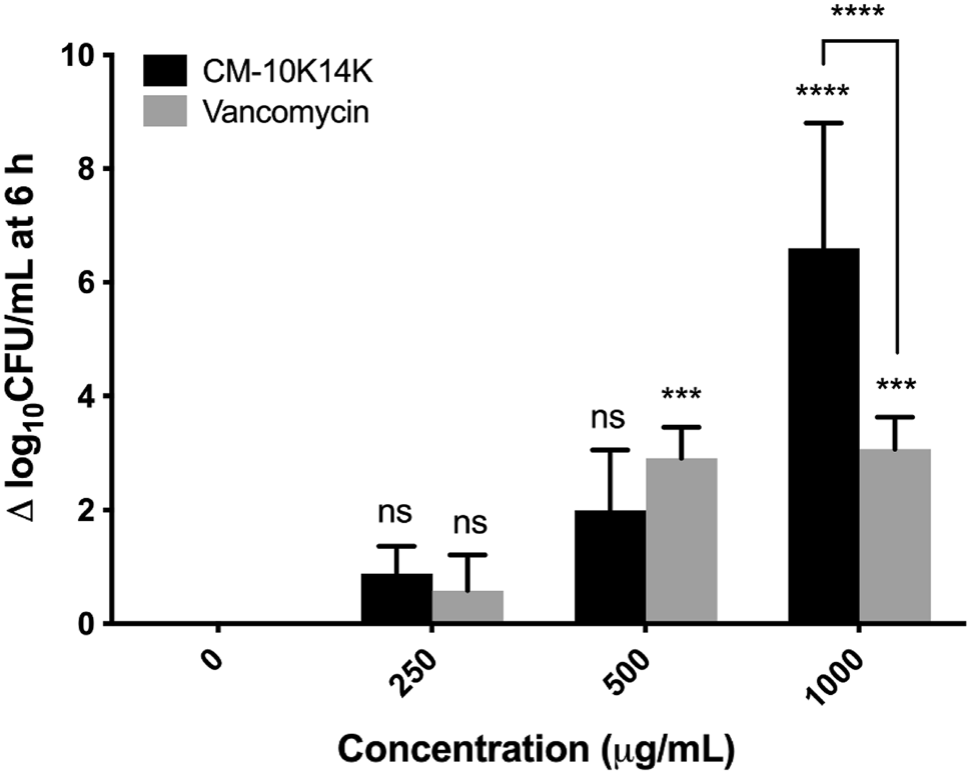
The bactericidal effect of CM-10K14K-impregnated catheters against planktonic *S. epidermidis* after 6 h of incubation compared to that of vancomycin. *P*-values were determined using a two-way ANOVA with Tukey’s post hoc test. Significant differences are indicated in GP style: > 0.05 (ns), ≤ 0.05 (*), ≤ 0.01 (**), ≤ 0.001 (***), ≤ 0.0001 (****).

The effect of peptide-impregnated catheters was evaluated in three ways: bactericidal activity against free-floating (planktonic) *S. epidermidis*, suppression of biofilm development by surviving bacteria, and inhibition of biofilm formation adherent to the catheter surface. Figure 14 shows the in vitro antibacterial activity of CM-10K14K-impregnated catheters compared with that of unmodified catheters; the assay allowed *S. epidermidis* colonization with bacterial cell counts ranging up to 10^7^–10^8^ CFU/mL. CM-10K14K catheters impregnated by dipping in 250, 500 or 1000 µg/mL had reduced bacterial counts of approximately 1 log_10_CFU/mL, 2 log_10_CFU/mL and 7 log_10_CFU/mL**,** respectively, after exposure to *S. epidermidis *in vitro for 6 h, and had significantly higher bactericidal activity than vancomycin-coated catheters at 1000 µg/mL dipped condition (Fig. 14). Although some bacterial cells survived and grew, CM-10K14K-impregnated catheters still displayed bactericidal activity 24 h after incubation (Supplementary Fig. 3).

**Figure 15:**
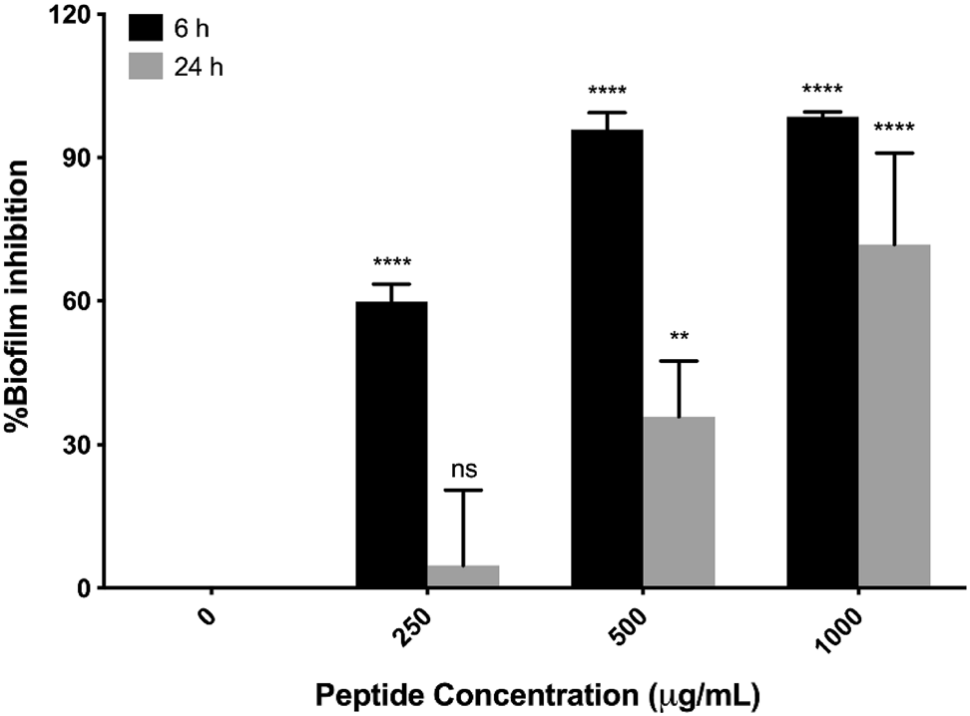
Biofilm inhibition on 24-well plate by CM-10K14K-impregnated catheters dipped in 250, 500 or 1000 µg/mL peptide and measured using the crystal violet assay. *P* values were determined using a two-way ANOVA with Tukey’s post hoc test. Significant differences are indicated in GP style: > 0.05 (ns), ≤ 0.05 (*), ≤ 0.01 (**), ≤ 0.001 (***), ≤ 0.0001 (****).

The CM-10K14K-impregnated catheters also inhibited biofilm formation by adherent cells on both 24-well plates and catheters in a dose-dependent manner when exposed to *S. epidermidis* for 6 and 24 h. On a 24-well plate, the impregnated CM-10K14K on catheters were released and diminished the biomass of *S. epidermidis* biofilms by more than 50% at 250 µg/mL. It reached to 90% at 500 and 1000 µg/mL dipped conditions after 6 h of incubation, as well as approximately 5%, 35% and 71% of biofilm inhibition after 24 h of treatment (Fig. 15).

**Figure 16:**
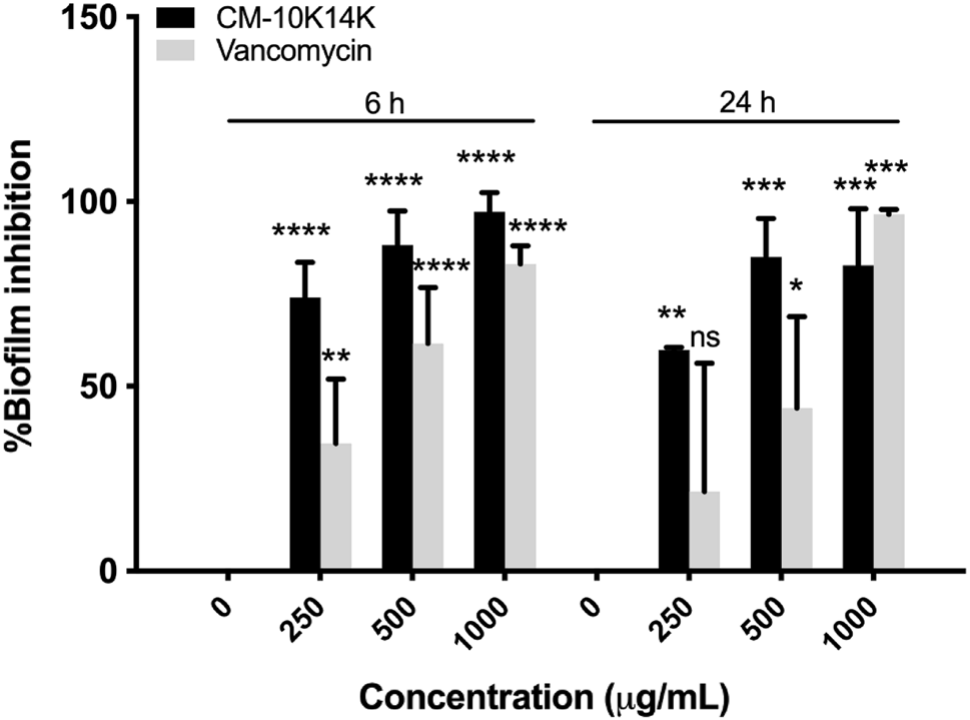
Inhibition of biofilm on CM-10K14K-impregnated catheters dipped in 250, 500 or 1000 µg/mL of peptide measured using the crystal violet assay. *P* values were determined using a two-way ANOVA with Tukey’s post hoc test. Significant differences are indicated in GP style: > 0.05 (ns), ≤ 0.05 (*), ≤ 0.01 (**), ≤ 0.001 (***), ≤ 0.0001 (****).

Biofilm formation on CM-10K14K-impregnated catheters was found to be decreased in a dose-dependent manner. After 6 h of incubation, CM-10K14K on the impregnated catheter reduced the biomass of *S. epidermidis* biofilms by 74.06 ± 9.51%, 88.17 ± 9.31% and 97.22 ± 5.19% at 250, 500 and 1000 µg/mL dipped conditions, respectively (Fig. 16). This inhibition of biofilm formation was greater than that of vancomycin-impregnated catheters, especially in the 250 µg/mL dipped condition. These results supported the finding of inhibition of *S. epidermidis* biofilm formation by CM-10K14K-impregnated catheters in vitro.

## Discussion

One of the most prevalent nosocomial diseases in hospitals is catheter-associated urinary tract infection (CAUTI), mostly caused by cross-contamination from the urinary drainage bag or the skin^11^, especially by *S. epidermidis*^10,37^. CAUTIs are reported in 80% of patients who have indwelling urinary catheters and in 10–50% of patients who have short-term catheterization^10^. Indwelling catheters are very susceptible to infection as bacteria can adhere, colonize and multiply quickly on their surfaces and subsequently form biofilms^11,17^. Major consequences such pyelonephritis, bacteremia, septicemia and sepsis can occur if left untreated, contributing significantly to the morbidity and mortality linked to CAUTIs^10,38^. Antimicrobial peptides (AMPs), with their biocompatible, broad-spectral antibacterial and antibiofilm properties, have significant potential for surface antifouling^39^. Since they are more effective against drug-resistant pathogens than existing antibiotic treatments, they provide a promising approach for the development of novel antimicrobial catheters to prevent CAUTIs.

Among the promising AMPs, 15-residue CM peptides [hybrids of the first seven amino acids of cecropin A and residues 2–9 of melittin, C(1–7)M(2–9)] in particular have attractive antimicrobial and antibiofilm properties^20,40^. In this study, the secondary structure and helical wheel projections of the CM parental peptide were exploited to design derivatives by amino acid substitution with potential antibacterial and antibiofilm activity along with low toxicity. The balance between positive charge and hydrophobicity is evidently important for the activity of short AMPs^41^ and thus in design strategy. Consistent with previous studies, our results indicate that lysine-substituted derivatives with increased net positive charge (CM-10K and CM-10K14K) had improved antibacterial and decreased hemolytic activities^40^. In contrast, derivatives with increased hydrophobicity (by substituting valine and alanine) did not have enhanced antibacterial activity. When derivatives with the same net charge and hydrophobicity were compared, those with the higher mean hydrophobic moment demonstrated lower hemolytic activity (greater HC_50_ values), such as CM versus CM-1V14K and CM-10K versus CM-1V10K14K. These results indicated that increased amphipathicity of peptides was associated with reduced toxicity against hRBCs^42^. Additionally, peptides with a lysine substitution at position 10 had significantly enhanced antibacterial and antibiofilm activity, pointing to the potential impact of increased net charge and a proper hydrophilic part on the polar face of the helical structure^41^. Antibiofilm activity was correlated with bactericidal activity of CM derivatives.

The capability of CM-10K14K to inhibit biofilm formation was most likely a result of the rapid killing of *S. epidermidis* bacteria in the initial inoculum through the direct antibacterial activity of the peptide. This suggested that the prevention of biofilm formation by CM-10K14K is primarily a consequence of its ability to promptly eliminate the initial bacterial population, thereby prevent biofilm development. Temporin 1Tb (TB) and its analogs (TB_KKG6A and TB_L1FK) were found to inhibit biofilm formation of *P. aeruginosa* and *S. aureus* at concentrations higher to their MIC^43^. The antibiofilm effect of these peptides is more likely attributed to the direct killing of biofilm-forming bacteria during their planktonic stage, similar to the mechanism observed with CM-10K14K, rather than acting through a biofilm-specific mechanism.

The eradication of mature biofilms presents a significant obstacle in the field of biofilm treatment, since up to 90% of an established biofilm is composed of an extracellular matrix. This matrix surrounds the bacteria and delays or prevents the penetration of antibiofilm agents into the biofilm structure^43^. Previous studies have emphasized the potential of peptide in combatting biofilms due to their amphipathic property and flexible molecular structure, allowing them to penetrate the extracellular matrix, prevent, and disrupt biofilms^44^. According to the findings of this study, CM-10K14K exhibited the capability to eliminate established *S. epidermidis* biofilms. However, a higher concentration of the peptide was necessary for this eradication compared to the concentration required for preventing biofilm formation. This suggested that action of the peptide to eradicate established biofilm may be limited by the presence of extracellular matrix.

CM-10K14K demonstrated more tolerant to proteinase K when compared to that of CM. The replacement of alanine and valine at positions 10 and 14 with two lysine residues enhanced the stability to proteinase K because this enzyme cleaves peptide bonds next to the carboxyl-terminal of aromatic and hydrophobic residues^45^. However, the antibacterial activity of CM-10K14K might be decreased in the higher concentration and/or other types of proteolytic enzyme, because of their sequence serve as excellent substrates for proteolytic cleavage. D-amino acid replacements might increase the biostability of CM-10K14K without reducing its antimicrobial activity because non-natural amino acids are less likely to degrade and tend to be more stable towards proteolysis^28^.

CAUTIs are caused by free-floating bacteria or those encrusted in and producing biofilms on the surfaces of medical devices. It thus may be necessary that indwelling catheters possess their own antibacterial and antibiofilm properties^14^. The impregnation of CM-10K14K onto urinary catheters by physical adsorption, a simple and traditional method of immobilizing AMPs, was developed to shield Foley catheters from colonization by biofilm-forming bacteria and thereby prevent associated infections^46^. In this study, Foley catheters were dipped in 250, 500 or 1000 µg/mL of CM-10K14K and subsequently released peptide, generating concentrations of approximately 18, 23 and 81 µg/mL, respectively, as tested by a fluorescamine assay (Supplementary Fig. 4). The antibacterial and antibiofilm activities of CM-10K14K-impregnated catheters were further assessed using an in vitro catheter-associated, urinary tract infection model. The release of CM-10K14K from impregnated catheters decreased planktonic growth and inhibited biofilm formation by adherent cells in a dose-dependent manner when challenged with *S. epidermidis* for 6 and 24 h. The results revealed that impregnation of CM-10K14K prevented bacterial adherence to catheters, hence minimizing the formation of biofilms on them.

Because of inherent differences between the membrane composition and architecture of microbial and mammalian cells allowing selectivity by AMPs^47^, CM-10K14K with potent antibacterial and antibiofilm activities along with minimal toxicity against mammalian cells (L929 mouse fibroblasts and human red blood cells) was identified as a candidate peptide. The mechanism of action of antibacterial and antibiofilm peptides is traditionally considered to be their disruption of bacterial membranes^48^. The negatively charged surfaces of *S. epidermidis*^49^ attract the positively charged CM-10K14K peptide via electrostatic interactions, and induce secondary structural transformation and interactions with the hydrophobic part of bacterial membranes. Circular dichroism spectroscopy was utilized to evaluate the secondary structure of the peptide. By shifting two negative signals at 222 nm and 208 nm, and a positive signal at 193 nm, the CD spectra of CM-10K14K showed that it formed an α-helical structure under membrane-mimetic conditions as predicted by the in silico 3D model. Following structural alteration, CM-10K14K killed viable *S. epidermidis* within 15 min in a concentration- and time-dependent manner, suggesting that the bacteria were killed even before they had the ability to divide because the generation times of surface growing *S. epidermidis* ranged from 17 to 38 min^50^. This is a crucial mechanism for preventing development of bacterial biofilms after initial attachment, as the survival of all other cells inside a biofilm is dependent on the interactions between the surface and the bacterial cells^51^.

AMPs typically kill bacteria by breaking their membranes or entering bacterial cells and interacting with internal components^52^. The membrane disruption mechanism was investigated by flow cytometry, SEM and TEM. Within 5 min, CM-10K14K induced both depolarization and permeabilization at the membrane level; within 15 min there was membrane damage with corrugation and distortion (seen by SEM) and membrane disintegration with dispersion of intracellular contents (seen by TEM). The strong affinity between the cationic peptide and anionic components of microbial pathogens leaded to rapid depolarization and permeabilization^53^. Most of membrane-lytic AMPs could rapidly destroy the bacterial membrane and some of them can kill bacteria in seconds after the initial contact with cell membrane^54^. In the opposite, some AMPs exhibited slow bactericidal action by exerting intracellular inhibitory effects via interacting at the molecular level, serving as the supportive mechanisms to achieve efficient killing^55^. Indolicidin induced increased permeabilization, facilitating continuous peptide entry into the cytosol to inhibit DNA replication and transcription^56,57^. Buforin II exhibited high nucleic acid binding affinity, implying that it inhibited cellular processes by interrupting DNA^55,58^. The antibiofilm activity of CM-10K14K was supported by SEM results which demonstrated a decrease in slimy extracellular matrix or biofilm as compared to untreated controls. Beside membrane-active mechanisms, the interaction of CM-10K14K with intracellular targets like DNA was investigated by gel retardation assay. CM-10K14K bound to genomic DNA of *S. epidermidis*, a possible target after penetration of the bacterial cell membrane and might play a part in the antibacterial activity. Due to its rapid bactericidal activity and multifunction, CM-10K14K might serve as the potential lead peptides for further development as novel antibacterial agents with improved therapeutic profiles.

To continue the clinical application of CM-10K14K as antimicrobial catheter, several factors including toxicity and stability should be concerned. A variety of coating and nanoparticle drug delivery system was optimized for each peptide to control the release, protect the peptide, and eventually overcome these obstacles. For example, functionalization of polyurethane catheters with peptide E6-brush coating can reduce bacterial adhesion on the catheter surface, inhibit planktonic bacterial growth in urine, and are biocompatible with bladder epithelial and fibroblast cells^17^. Srisang et al. revealed that chlorhexidine-loaded nanoparticles (CHX-NPs) coated catheters demonstrated delayed encrustation and bacterial colonization. Furthermore, in vivo biocompatibility experiments revealed that coated catheters did not induce dermal toxicity^59^. Further development of the drug delivery or coating strategy as well as the study of underlying antibiofilm mechanism at molecular level and in vivo efficacy could enhance the clinical feasibility of CM-10K14K.

### Supplementary Information

Supplementary Information.

## Data Availability

All of the data produced throughout the research are contained in this article and its supplementary information file.
