# Supplementary material for: The bactericidal and antibiofilm effects of a lysine-substituted hybrid peptide, CM-10K14K, on biofilm-forming Staphylococcus epidermidis
Source: Sci Rep. 2023 Dec 14;13:22262. doi: 10.1038/s41598-023-49302-y (PMC10721899; doi:10.1038/s41598-023-49302-y)
Supplement: Supplementary file 1 — Supplementary Information. [file 41598_2023_49302_MOESM1_ESM.docx]

**Supplementary Information**

**The bactericidal and antibiofilm effects of a lysine-substituted hybrid peptide, CM-10K14K, on biofilm-forming *Staphylococcus epidermidis***

Natthaporn Klubthawee^1^, Mathira Wongchai^2^ and Ratchaneewan Aunpad^2,*^

^1^Department of Medical Technology, Faculty of Allied Health Sciences, Thammasat University, Pathum Thani, 12120, Thailand.

^2^Graduate Program in Biomedical Sciences, Faculty of Allied Health Sciences, Thammasat University, Pathum Thani, 12120, Thailand.

*Corresponding author

Email: aratchan@tu.ac.th; +66829869213

**1. Biofilm inhibition assay**

The biofilm biomass was assessed using crystal violet staining to determine the ability of peptides to inhibit bacterial formation of biofilm ^1^. Overnight *S. epidermidis* cultures were adjusted to an OD620 of 0.1 in TSB. An equal volume (50 µL) of bacterial suspension (~10^8^ CFU/mL) was incubated with 2-fold serially diluted peptides (0.98-250 μg/mL) at 37°C for 24 h in 96-well plates. Supernatants were discarded after incubation, and wells were gently rinsed at least twice with 0.85% NaCl to remove planktonic cells. Adherent biofilms were fixed for 30 minutes with 95% ethanol, then the fixative solution was removed. The fixed biofilms were dried thoroughly and stained with addition of 150 µL of 0.1% crystal violet (CV) to each well containing biofilm, along with its respective control media. After 15 min, CV solutions were removed and wells were gently rinsed three times with 400 μL of 0.85% NaCl without disturbing the biofilm. The plates were left to dry for 30 min before adding 200 μL of 95% ethanol to each well to solubilize the bound CV with vigorous pipetting. The biofilm mass was evaluated by observing OD at 570 nm using a Multiskan SkyHigh microplate reader ^2^. Results were expressed as the percentage of biofilm mass compared to that of control.

The antibiofilm activity of designed peptides compared to that of vancomycin, the results showed that all peptides were found to inhibit *S. epidermidis* biofilm formation in a concentration-dependent manner. After treatment with > 15.63 µg/mL for 24 h, each peptide and vancomycin reduced the biomass of biofilms by more than 90% (Supplementary Figure 1). Thus, 15.63 µg/mL was used to represent the biofilm-forming inhibiting concentration ^2^. Interestingly, all derivatives, even peptides with slightly lower antibacterial activity (CM-1V10K14K, CM-1V14K and CM-A), showed significantly higher antibiofilm activities than did the parental peptide at their respective MICs (3.91-7.81 µg/mL). In contrast, vancomycin slightly induced biofilm formation at its MIC and MBC (Supplementary Figure 1). Among all derivatives, CM-1V10K14K, CM-10K and CM-10K14K showed the most potent biofilm inhibition, followed by CM-A, CM-1V14K and the parental peptide.


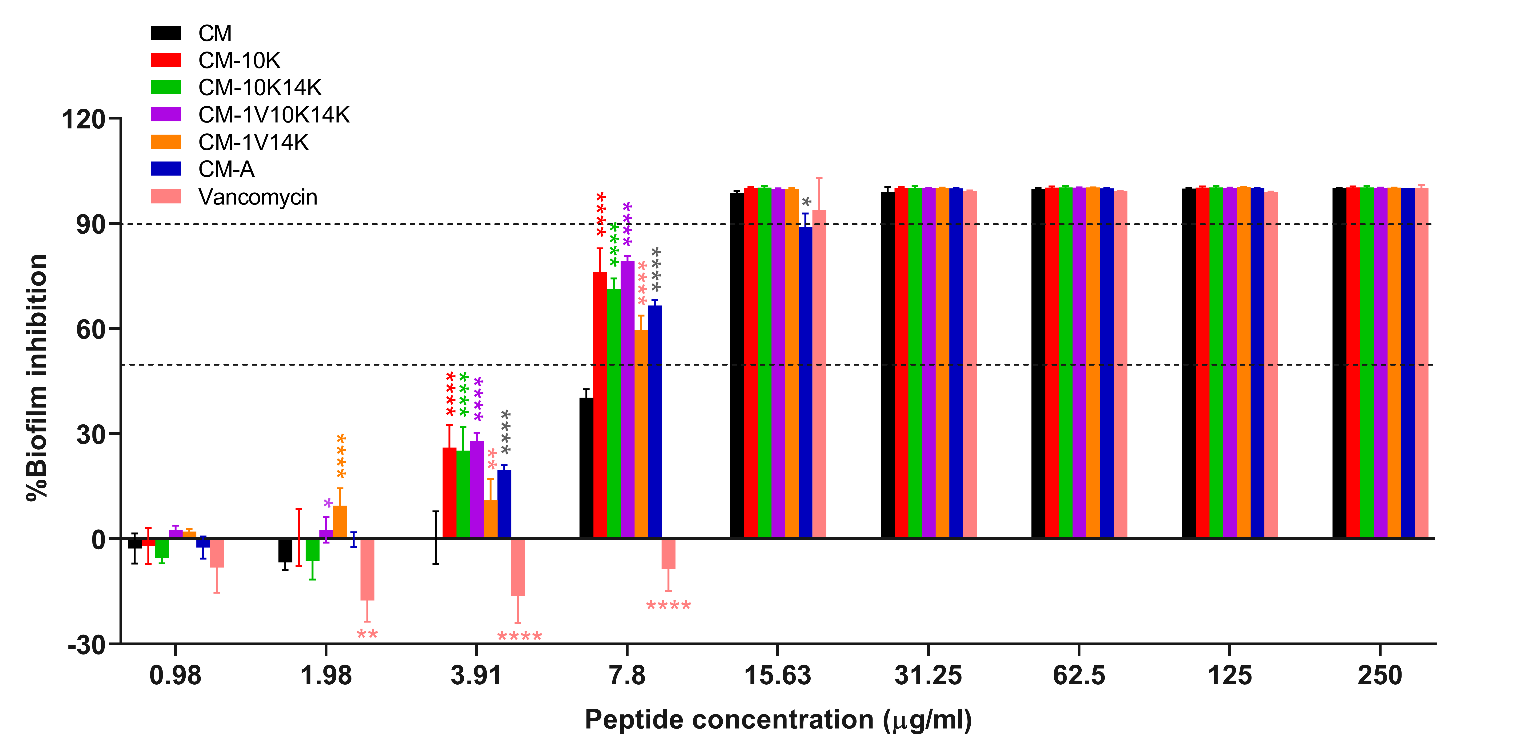


**Supplementary Figure 1.** Biofilm-inhibition of CM derivatives compared to vancomycin against *S. epidermidis* ATCC 35984. The experiments were performed in triplicate and the data were expressed as the mean ± SD. *p*-values were determined using a two-way ANOVA with Tukey’s post hoc test. Significant differences are indicated in GP style: >0.05 (ns), ≤0.05 (*), ≤0.01 (**), ≤0.001 (***), ≤0.0001 (****).

**2. Analysis of the bacterial DNA-binding activity of CM-10K14K using a gel retardation assay.**

**
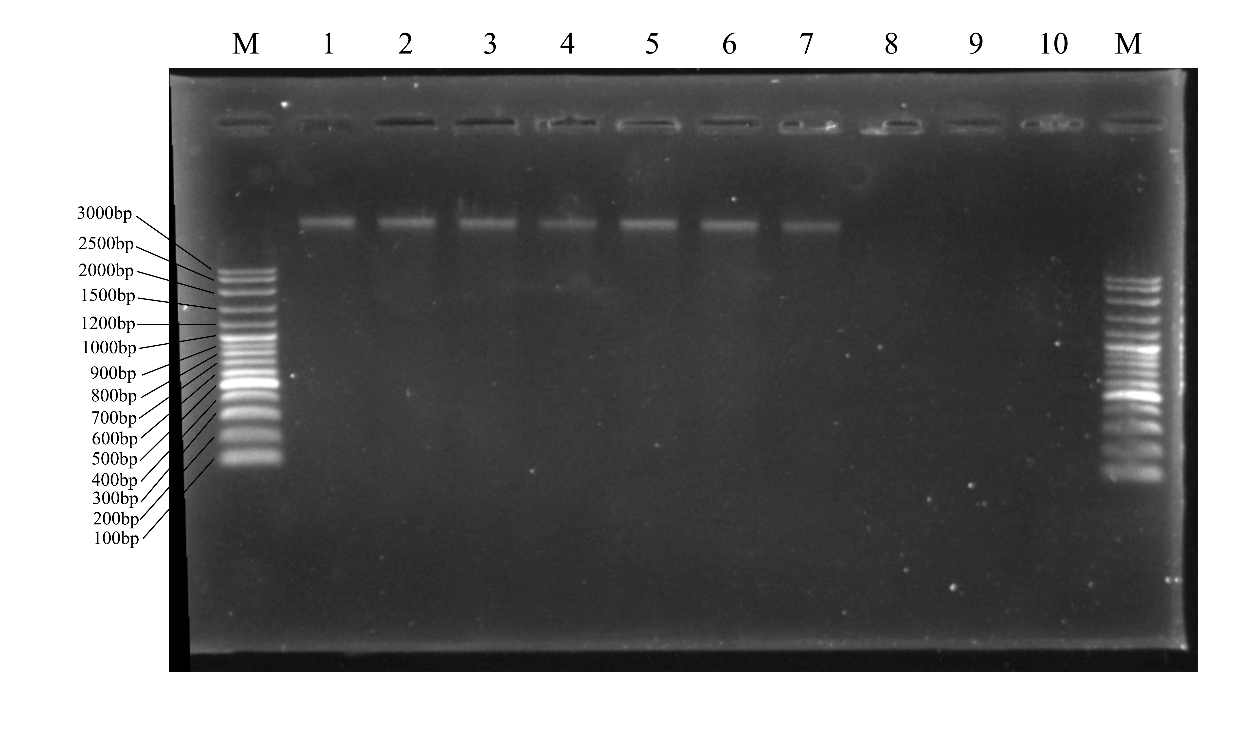
**

**Supplementary Figure 2.** The original gel of a gel retardation assay.

**3. Determination of viable planktonic cells by colony count assay**


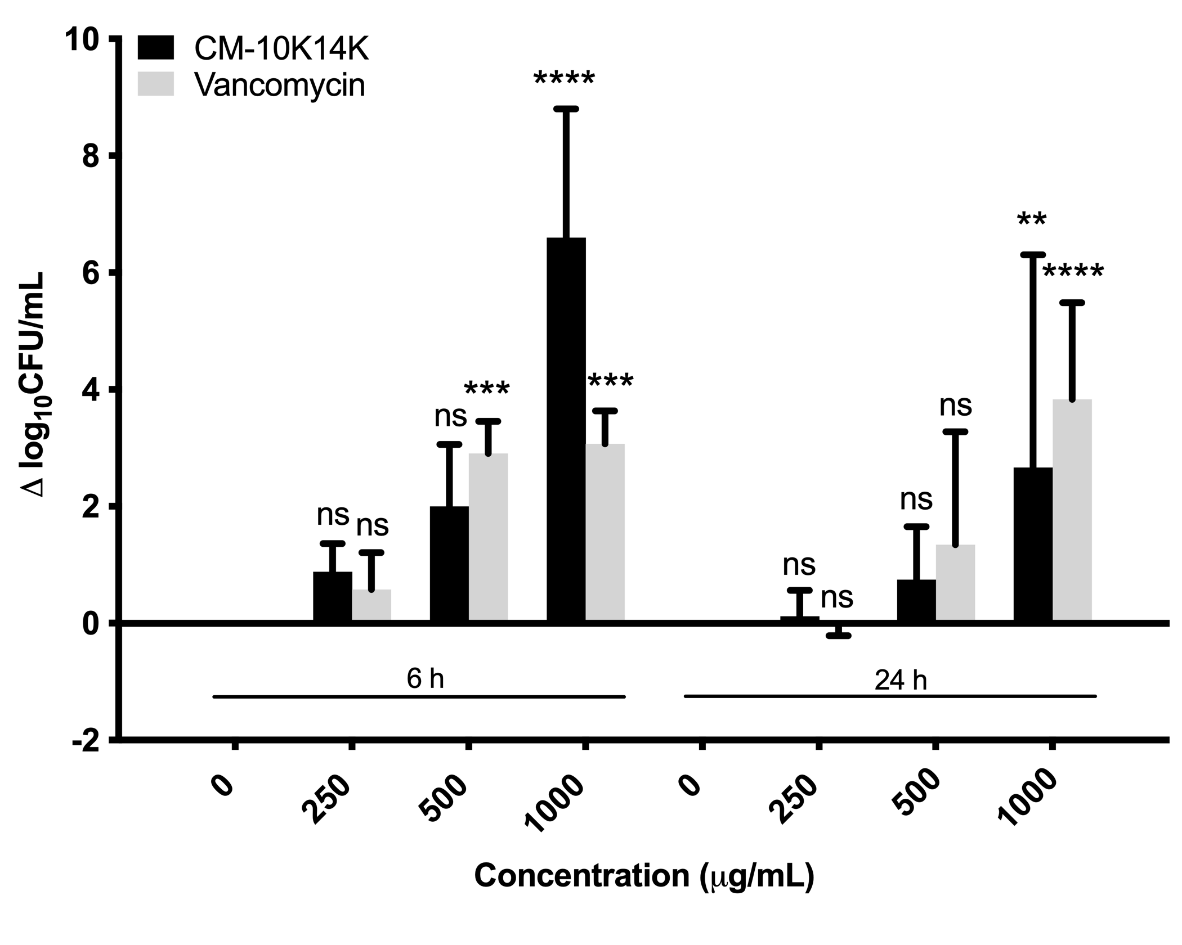


**Supplementary Figure 3.** The bactericidal effect of CM-10K14K-impregnated catheters against planktonic *S. epidermidis* ATCC 35984 after 6 h and 24 h of incubation compared to that of vancomycin. *p*-values were determined using a two-way ANOVA with Tukey’s post hoc test. Significant differences are indicated in GP style: >0.05 (ns), ≤0.05 (*), ≤0.01 (**), ≤0.001 (***), ≤0.0001 (****).

**3. Quantification of impregnated peptide by fluorescamine assay**

The release of peptide from impregnated catheter was determined by quantifying CM-10K14K after preparation. The dipped catheters with 250, 500 and 1000 µg/mL of CM-10K14K solutions were prepared following Fig.2. Each segment of dipped catheters was incubated with 200 µL of ultrapure water at room temperature for 24 hours. The fluorescamine assay was used to detect the released CM-10K14K in ultrapure water according to the manufacturer's instructions. The peptide's primary amines react with fluorescamine to create fluorescent products that can be measured using fluorescence spectroscopy. In short, 3 mg of fluorescamine was dissolved in 1 mL DMSO. Fifty µL of the fluorescamine solution was mixed with 150 µL of the released peptide and then allowed to incubate at room temperature for 15 min in the dark. Using a fluorescent microplate reader, the fluorescent signal (excitation: 355/40 nm, emission: 460/20 nm) was quantified. To generate a standard curve, the reaction mixture of two-fold serial dilution of CM-10K14K (concentration ranging from 1.95 to 125 µg/mL) was performed (Supplementary Figure 3). The amount of CM-10K14K in the sample supernatants were compared to the standard curve. All measurements were performed in triplicate.


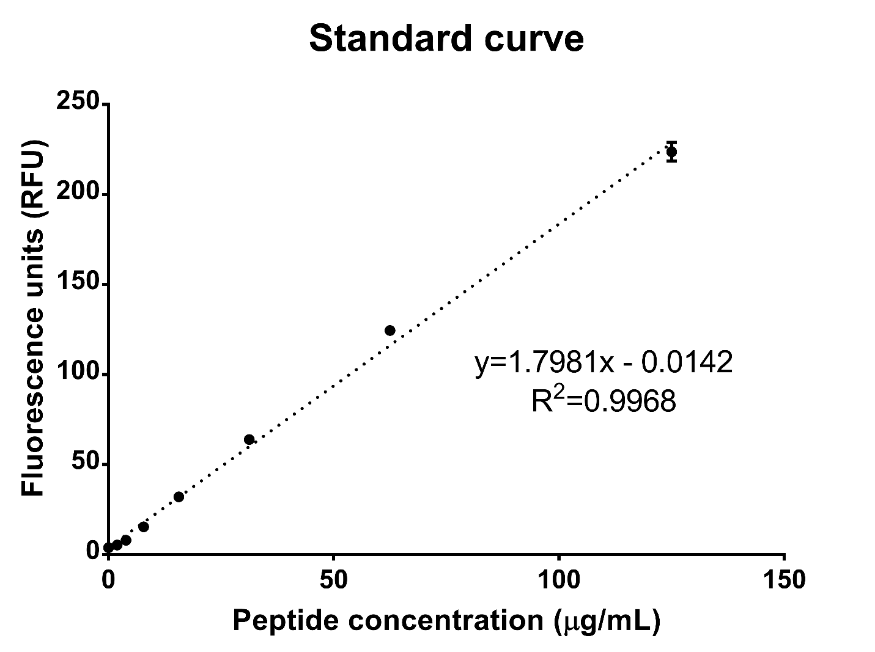


**Supplementary Figure 3.** The standard curve of CM-10K14K by fluorescamine assay.

**Supplementary Table 1.** The potential cleavage sites of CM and CM-10K14K sequence, cleaved by proteinase K, predicted by PeptideCutter online program.

| **CM** | | **CM-10K14K** | |
| --- | --- | --- | --- |
| **Position of**  **cleavage site** | **Resulting**  **peptide sequence** | **Position of**  **cleavage site** | **Resulting**  **peptide sequence** |
| 2 | KW | 2 | KW |
| 4 | KL | 4 | KL |
| 5 | F | 5 | F |
| 8 | KKI | 8 | KKI |
| 10 | GA | 11 | GKV |
| 11 | V | 12 | L |
| 12 | L | 15 | KKL  (end of sequence) |
| 14 | KV |  |  |
| 15 | L  (end of sequence) |  |  |

References

1. Kim, M. K., Kang, H. K., Ko, S. J., Hong, M. J., Bang, J. K., Seo, C. H., & Park, Y. (2018). Mechanisms driving the antibacterial and antibiofilm properties of Hp1404 and its analogue peptides against multidrug-resistant *Pseudomonas aeruginosa.* *Scientific Reports, 8*(1), 1763. doi:10.1038/s41598-018-19434-7

2. Cruz, C. D., Shah, S., & Tammela, P. (2018). Defining conditions for biofilm inhibition and eradication assays for Gram-positive clinical reference strains. *BMC Microbiology, 18*(1), 173. doi:10.1186/s12866-018-1321-6
